# Integrative functional genomics reveals transcriptional regulatory function of risk alleles for metabolic liver disease

**DOI:** 10.21203/rs.3.rs-6984670/v1

**Published:** 2025-10-23

**Authors:** Wenxiang Hu, Biying Zhu, Na He, Yang Xiao, Bin Chen, Chen Li, Ravi Mandla, Yifan Liu, Jiayu Zhang, Xiao Chang, Fulong Yu, Marijana Vujkovic, Julie Lynch, Kyong-Mi Chang, VA Million Veteran Program, Bogdan Pasaniuc, Daniel Rader, Mitchell A. Lazar

**Affiliations:** Guangzhou National Laboratory; Guangzhou National Laboratory; Guangzhou National Laboratory; University of Pennsylvania; Guangzhou National Laboratory; Guangzhou National Laboratory; University of Pennsylvania; University of Pennsylvania; University of Pennsylvania; Shandong First Medical University; Guangzhou National Laboratory; University of Pennsylvania School of Medicine; Salt Lake City VA Healthcare System; The Corporal Michael J. Crescenz Veterans Affairs Medical Center and University of Pennsylvania Perelman School of Medicine; U.S Department of Veteran Affairs; University of Pennsylvania; University of Pennsylvania; University of Pennsylvania

## Abstract

Genome-wide association studies (GWAS) have identified nearly 100 loci associated with metabolic dysfunction-associated steatotic liver disease (MASLD), but the molecular functions of these variant alleles remain elusive, particularly when they occur in non-coding regions. Here we profiled the chromatin accessibility landscape of liver nuclei from MASLD individuals, and demonstrated these accessible genomic sites were bound by cell type-specific transcription factors (TFs) and enriched for MASLD risk variants, highlighting lineage- and disease state-specific regulation. Using a massively parallel reporter assay (MPRA), we identified hundreds of differential activity variants (DAVs) that operate in a cell type-specific manner or in a stimulus-dependent context by disrupting liver pathogenesis-associated transcriptional regulatory network. Integrative analyses combining liver eQTLs, chromatin looping, and single-cell CRISPRi screening linked these DAVs to functional target genes. Notably, we demonstrated that DAVs located near *SLC22A3* and core regulators of triglyceride metabolism (*APOA5*, *ANGPTL3*, and *LPL*) loci modulate their gene expression and contribute to altered lipid metabolism and hepatic stellate cell activation. Furthermore, these DAVs exhibit predictive power in distinguishing MASLD disease risk. Together, these multimodal integration analyses provide insights into the regulatory mechanisms of MASLD progression driven by noncoding genetic risks.

## Introduction

Metabolic dysfunction-associated steatotic liver disease (MASLD), the most common chronic liver condition, encompasses a spectrum from metabolic dysfunction-associated steatotic liver (MASL), characterized by simple lipid accumulation, to advanced metabolic dysfunction-associated steatohepatitis (MASH), marked by progressive inflammation and fibrosis^1^. Given the limited therapeutic options for MASLD, there is an urgent need to elucidate its underlying causal mechanisms. MASLD arises from the complexed interplay between environmental factors and genetic predisposition, with heritability estimates ranging from 20% to 70%^2^. Large-scale genome-wide association studies (GWAS) have identified nearly 100 loci associated with MASLD risk, but most variants lie in noncoding regions with unknown functions^3–7^. Furthermore, high linkage disequilibrium (LD) and genetic pleiotropy complicate the identification of causal variants and target genes. Numerous computational methodologies, including fine-mapping, expression quantitative trait loci (eQTL) analysis, chromatin accessibility quantitative trait loci (caQTL) analysis, and deltaSVM algorithms, have been employed to identify causal functional variants, but they have demonstrated limited predictive power^8–10^.

Noncoding genetic variants can influence gene expression by altering chromatin accessibility and transcription factor binding at gene regulatory elements (GREs), such as enhancers^11,12^. Massively parallel reporter assays (MPRA) offer a scalable experimental approach to test the enhancer activity of risk alleles in disease-relevant cell types, organoids, or organisms under various environmental conditions^13–15^. Since GREs are often cell-type-specific, it is crucial to understand the role of disease-associated variants within relevant cell types. While many studies using single-cell/nuclei RNA sequencing (scRNA-seq or snRNA-seq) have shown that MASLD pathophysiology involves multiple liver cell types^16,17^, the chromatin landscape heterogeneity in MASLD patient livers has been rarely explored. This understanding is essential for interpreting the molecular mechanisms underlying noncoding MASLD risk variants.

Here, we profiled the chromatin accessibility landscape of liver nuclei from MASLD patients and found that accessible regions were enriched for MASLD risk variants and bound by cell type-specific transcription factors. MPRA screening identified hundreds of differential activity variants (DAVs) with context-specific regulatory activity that perturb transcriptional networks associated with liver pathogenesis. Integration with liver eQTL data, chromatin interaction maps, and single-cell CRISPRi screening enabled the assignment of target genes to these regulatory variants. Notably, we validated key DAVs that modulate lipid metabolism and drive hepatic stellate cell activation, the hallmarks of MASLD pathology. Importantly, these DAVs exhibit predictive power in distinguishing MASLD disease risk. Thus, our multimodal analyses reveal how noncoding genetic variation drives MASLD progression through disruption of cell type- and state-specific regulatory programs.

## Results

### Cell type-specific regulation of chromatin accessibility by MASLD risk variants

**Figure 1:**
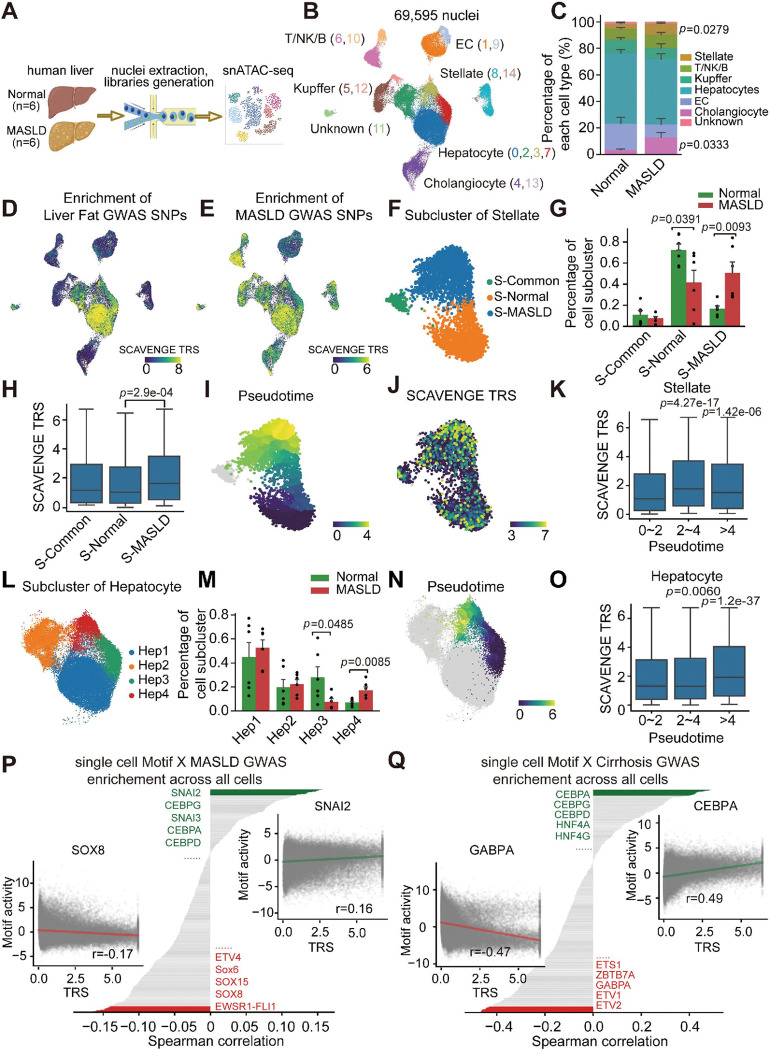
Cell type-specific accessible chromatin in the liver enriches for MASLD risk variants. **(A)** Schematic illustration of snATAC-seq in normal and MASLD livers. **(B)** Fully integrated UMAP of 69,595 nuclei from human livers identifies 15 distinct clusters labeled 0–14. **(C)** Comparison of cell type proportions between normal and MASLD liver samples. Bar plots show the relative abundance of each annotated cell population under the two conditions (n = 6). **(D-E)** UMAP visualization of per-cell Trait Relevance Scores (TRSs) calculated by SCAVENGE for the liver fat trait (D) and MASLD trait (E). Cells are colored according to their TRS, highlighting trait-associated cell populations. **(F-G)** UMAP visualization of three distinct hepatic stellate cell subclusters (F) and their relative proportions across normal and MASLD conditions (G). **(H)** Box plots illustrating SCAVENGE TRS scores for MASLD traits across the three stellate cell subclusters. Boxes represent the interquartile range (IQR), horizontal lines indicate the median, and whiskers denote 1.5× IQR. **(I)** Pseudotime trajectory overlaid on the UMAP of hepatic stellate cells, illustrating the inferred dynamic transition states among subclusters. **(J-K)** UMAP (J) and box plots (K) illustratingSCAVENGE TRS scores for MASLD traits across the stellate cell activation trajectory. Boxes represent the interquartile range (IQR), horizontal lines indicate the median, and whiskers denote 1.5× IQR. **(L-M)** UMAP visualization of four distinct hepatocyte subclusters (L) and their relative proportions across normal and MASLD conditions (M). **(N-O)** UMAP (N) and box plots (O) illustrating SCAVENGE TRS scores for MASLD traits along the hepatic remodeling trajectory. Boxes represent the interquartile range (IQR), horizontal lines indicate the median, and whiskers denote 1.5× IQR. **(P-Q)** Correlation of TF motif enrichment with MASLD- (P) and Cirrhosis-associated risk variants (Q) enrichment in all cells. TF motif activity for each cell was quantified using chromVAR, and TRS for individual cells were computed using SCAVENGE. Spearman’s rank correlation coefficients were then calculated between motif activity and TRS across all cells to assess the association between transcription factor activity and GWAS trait enrichment.Top 5 motifs were shown. Data are presented as mean ± SEM for proportion analyses shown in panels (C), (G), and (M). Statistical significance was determined using two-tailed Student’s t-test.

To delineate the regulatory program of MASLD-associated genetic noncoding variants, we profiled the chromatin landscape of MASLD patient liver. We performed single-nucleus ATAC sequencing (snATAC-seq) on liver nuclei from 12 individuals (6 Normal and 6 MASLD) (Fig. 1A), and identified 69,595 high-quality nuclei and classified them into 15 distinct cell clusters (Fig. 1B **and Extended Data** Fig. 1A-1K). Each cluster displayed a unique chromatin accessibility landscape and TF motif enrichment signature, reflecting robust cell identity-specific regulatory programs (**Extended Data** Fig. 2A-2C). Our cell type annotations were cross-validated using a recently published human liver snATAC reference dataset (**Extended Data** Fig. 2D-2E)^18^. Furthermore, by inferring the gene scores of marker genes of each cluster (**Extended Data** Fig. 2F), we identified clusters representing hepatocytes (*ALB*), cholangiocytes (*KRT7*), Kupffer cells (*CD163*), stellate cells (*NGF*), endothelial cells (*FLT1*), and T/NK/B cells (*CD8A*), with each cell cluster expressing a unique transcriptional program (Fig. 1B **and Extended Data** Fig. 2G). We observed highly specific correlations between marker genes defined by snATAC-seq and previous snRNA-seq from human MASLD liver (**Extended Data** Fig. 2H)^17^. As expected, MASLD liver samples consisted of higher percentages of cholangiocytes and stellate cells, and decreased hepatocyte and endothelial cells, compared to normal livers (Fig. 1C **and Extended Data** Fig. 1H-1J)^19^.

We next sought to test whether MASLD-associated variants were enriched in liver regulatory elements. Stratified linkage disequilibrium score regression (LDSC) analysis demonstrated enrichment of MASLD GWAS variants in hepatocyte and stellate cell chromatin peaks, while plasma triglyceride trait-associated variants were enriched in accessible genomic regions of hepatocytes and Kupffer cells (**Extended Data** Fig. 3A-3B). In contrast, no enrichment was observed for variants associated with body mass index (BMI) traits. To further investigate the heterogeneity of genetic association enrichment, we applied SCAVENGE, an algorithm we developed that integrates scRNA-seq and GWAS data to identify disease-associated genetic variants at single-cell resolution^20^. Our analysis revealed that liver fat trait-associated variants were specifically enriched in hepatocyte peaks, whereas MASLD-associated variants displayed a broader pattern of enrichment across various cell types, including hepatocytes, stellate cells, and Kupffer cells (Fig. 1D–1E), suggesting the genetic involvement of non-parenchymal cells in addition to hepatocytes in MASLD.

We then focused on stellate cells, the primary effector cells in MASLD progression^21^, and identified three sub-clusters with varying chromatin accessibility. Based on the cluster composition of cell origin, we termed these three stellate cell clusters as S-MASLD, S-Normal, and S-Common (Fig. 1F–1G). The proportion of S-MASLD cells was higher in MASLD liver compared to normal liver. Genomic region enrichment analysis of S-MASLD-specific peaks revealed pathways related to extracellular matrix organization and inflammatory responses (**Extended Data** Fig. 3C-3D). Strikingly, MASLD genetic risk variants were differentially enriched among stellate subtypes, with S-MASLD cells exhibiting the highest SCAVENGE trait relevance scores (TRS) (Fig. 1H). Pseudotime trajectory analysis, validated by two independent algorithms, revealed a continuous activation process across stellate cell states, during which MASLD risk variants became progressively enriched (Fig. 1I–1K **and Extended Data** Fig. 3E-3F). Beyond stellate cells, we observed substantial chromatin heterogeneity among hepatocyte subpopulations. Disease-enriched hepatocyte states showed preferential localization of MASLD-associated risk variants (Fig. 1L–1O **and Extended Data** Fig. 3G-3J). A similar trend was observed in Kupffer cells, where disease-associated Kupffer cells exhibited notably higher SCAVENGE TRS scores (**Extended Data** Fig. 4A-4F). Collectively, these findings indicate that MASLD risk variants exert cell type- and cell state-specific effects on the hepatic regulatory landscape to drive disease progression.

Given our ability to simultaneously map GWAS variant enrichments using SCAVENGE and TF motif activities using chromVAR at single-cell resolution, we next performed integrative analyses to gain mechanistic insight into how noncoding risk variants might influence transcriptional regulation in MASLD^10^. We observed a positive correlation between MASLD enrichment TRS scores and the motif activities of TFs related to metabolism and fibrosis, such as SNAI2, across all profiled nuclei (Fig. 1P)^22^. A similar trend was seen when the analysis was performed in hepatocytes alone, where CEBPA motif activities were positively correlated with MASLD trait TRS scores (**Extended Data** Fig. 4G). Additionally, integrative analysis of cirrhosis, a more advanced stage of MASLD, revealed the strongest positive correlations with TFs like CEBPA, HNF4A, and NR2F6, and negative correlations with TFs such as GABPA (Fig. 1Q **and Extended Data** Fig. 4H-4I), which is involved in oxidative phosphorylation^23^. To further dissect the regulatory architecture of MASLD progression, we integrated snRNA-seq and snATAC-seq data to construct hepatocyte-specific transcriptional regulatory networks using Pando^24^. We revealed extensive disease-dependent transcriptional regulatory network rewiring, characterized by altered expression and motif enrichment of key TFs implicated in MASLD pathogenesis, including FOXO3, MLXIPL, PROX1, PPAR, HNF1B/4A, and TEAD1 (**Extended Data** Fig. 4J-K).

### Identify and characterize the candidate functional variants using MPRA

**Figure 2:**
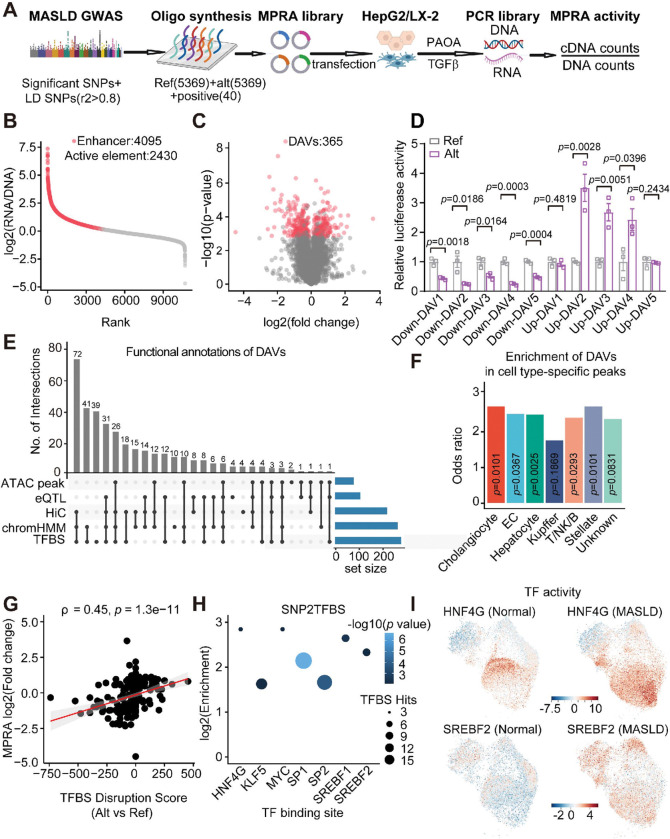
Identification and characterization of functional variants using MPRA. **(A)** Schematic overview of the MPRA experimental workflow. The assay was performed in four biological replicates per condition to ensure reproducibility of enhancer activity measurements. **(B)** Enhancer activity for each element was plotted according to its activity rank. Alleles were classified as Enhancers (red) if they had an adjusted *p*-value < 0.05 and a log2 fold change > 0. Active elements were transcriptionally active for at least one allele. **(C)** Volcano plot showing the effect sizes and *p* values of MPRA variants. Variants with significant (FDR < 0.01) allele-differential activity (DAVs) are highlighted in red dots. **(D)** The activities of luciferase reporters with the different alleles for ten random DAVs in HepG2. Data are shown as mean ± SEM fold change relative to their own ref allele (Student’s t test). **(E)** Intersection sizes of DAVs overlapping with functional annotations such as ATAC peaks from snATAC-seq data, liver eQTLs, liver chromatin interactions, transcription factor binding motif disruption, and ChromHMM states. **(F)** Enrichment of DAVs within liver cell type-specific accessible chromatin regions. Total tested variants were used as the background set. The *p* values were showed (Fisher’s exact test). **(G)** Correlation between DAV effect sizes and TF motif disruption scores, indicating functional consequences of allelic variants on TF binding. **(H)** The enrichment of transcription factor motifs affected by DAVs, as identified by the SNP2TFBS algorithm. **(I)** UMAP plots displaying the motif activity of DAV-affected TFs, including HNF4G and SREBF2, within normal and MASLD hepatocytes. Colors indicate normalized motif enrichment scores.

To systematically identify functional noncoding variants associated with MASLD, we designed an MPRA library that includes both alleles of 5,369 noncoding variants in strong linkage disequilibrium (r^2^ >0.8) with genome-wide significant as well as subthreshold significant variants from recent GWASs (**Extended Data** Fig. 5A **and Table S1**)^4,6,7,25–30^. Notably, we included subthreshold variants in the MPRA library based on their potential to modulate enhancer function and contribute to MASLD risk, despite not surpassing conventional significance thresholds for GWAS^31,32^. Interestingly, these MPRA candidate variants were predominantly enriched in regions of open chromatin within primary liver cell types, such as hepatocytes and immune cells (**Extended Data** Fig. 5B-5D). The MPRA was conducted in disease-relevant hepatic cell lines, specifically the hepatocyte cell line HepG2 and the stellate cell line LX-2, both with and without environmental stimuli (fatty acid and TGFβ) to simulate environmental risk factors associated with MASLD (Fig. 2A). The MPRA library was synthesized with a median library complexity of 714 barcodes per allele and included 40 positive regulatory elements predicted to be active in hepatocytes (**Extended Data** Fig. 5E). The positive regulatory elements demonstrated elevated transcriptional activity, whereas the negative regulatory elements exhibited minimal activity in HepG2 cells, validating the robustness of the MPRA approach (**Extended Data** Fig. 5F-5G). MPRA was performed with four replicates for each condition, and exhibited high reproducibility, as exemplified in HepG2 (**Extended Data** Fig. 5H). Overall, 2430 regulatory elements were transcriptionally active for at least one allele in HepG2 and enriched for metabolic TFs such as HNF4, CEBPD and TRβ (Fig. 2B **and Extended Data** Fig. 6A). We subsequently assessed allele-specific activity within these active elements, identifying 365 differential activity variants (DAVs) [FDR < 0.01] distributed across 21 chromosomes (Fig. 2C, **Extended Data** Fig. 6B-6C **and Table S2**). To validate the sensitivity and specificity of the DAVs identified by MPRA, we randomly selected 10 DAVs with high allele-specific activity and examined their activities individually. Notably, eight out of ten DAVs demonstrated the expected allele-specific activity in HepG2 cells (Fig. 2D), confirming the reliability of the MPRA.

We next characterized the properties of the DAVs through integrative analysis, incorporating chromatin accessibility information identified by snATAC-seq, liver eQTL data, liver or HepG2 chromatin interaction data, TF motif disruption, and liver chromatin functional annotations^33,34^. Nearly 90% of DAVs were located within active genomic regions, and 297 out of 365 DAVs overlapped with two or more functional annotations (Fig. 2E **and Extended Data** Fig. 6D-6E). Of particular interest were 87 DAVs that were located in chromatin-accessible regions across various cell types, with higher enrichment observed in open chromatin regions of hepatocytes, stellate cells, and cholangiocytes (Fig. 2F **and Extended Data** Fig. 6F-6H). For example, several DAVs located in the *GLDC* and *MLXIP* loci reside within hepatocyte-specific chromatin peaks and were associated with strong enhancer signals (**Extended Data** Fig. 7A-7B). Notably, 274 DAVs altered the motif activity of specific TFs. We observed a positive correlation between TF binding disruption scores and MPRA effect sizes for the DAVs (Fig. 2G). Specifically, HNF4G, SREBF1/2, and ETS (Erythroblast Transformation-Specific) family binding sites were enriched for DAVs, as determined using two different algorithms, SNP2TFBS and MotifbreakR (Fig. 2H **and Extended Data** Fig. 6I). These results are consistent with the established roles of HNF4 and SREBF1/2 in liver metabolism and underscore the involvement of ETS family transcription factors in MASLD progression^35–38^. Furthermore, we observed that motif activities of HNF4G and SREBF1/2 were higher in the MASLD liver compared to the normal liver, whereas the ETS family member GABPα binding activity was reduced in MASLD liver (Fig. 2I **and Extended Data Fig. 8A-8E**). Together, these functional annotations allow us to prioritize potential regulatory variants and nominate their target genes with greater precision.

### DAVs exhibit cell type-specific regulatory function

**Figure 3:**
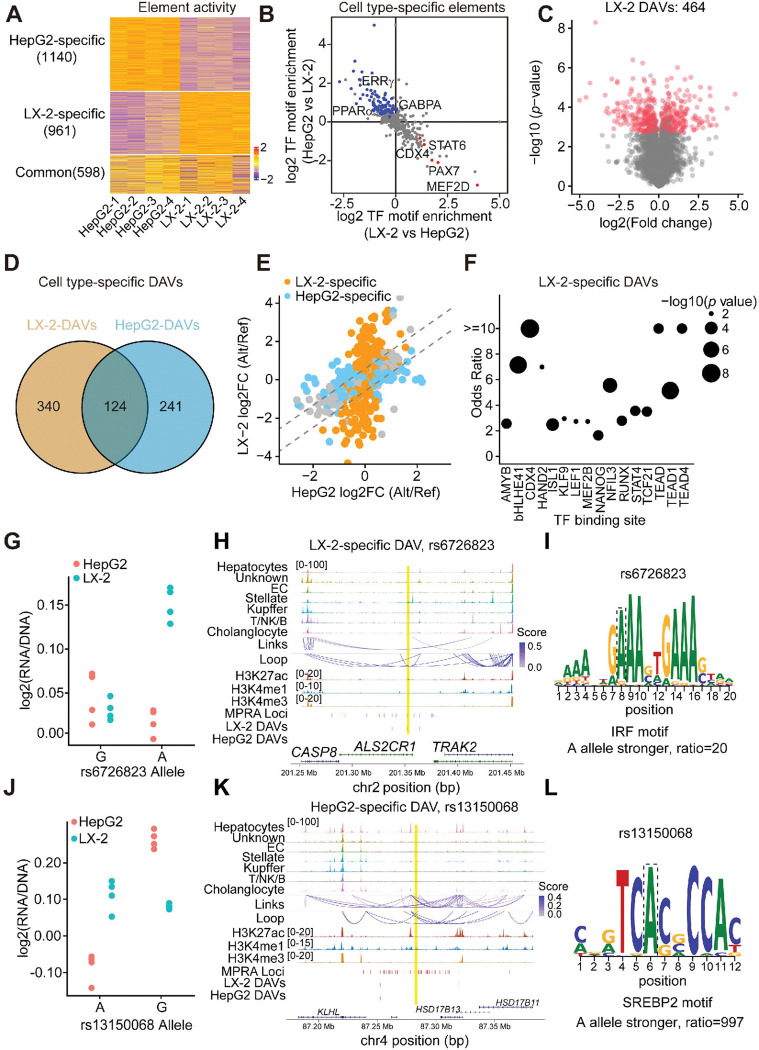
Identification of cell type-specific DAVs. **(A)** Heatmap of cell type-specific active elements significantly active in HepG2 or LX-2 cell lines. Differential element activity were identified with FDR < 0.05. Color scale represents the ratio of RNA/DNA in normalized counts. **(B)** TF motif enrichment analysis for cell type-specific active elements identified in HepG2 and LX-2. Colors indicate significantly enriched motifs (q < 0.1). **(C)** Volcano plot showing the effect sizes and *p* values of MPRA variants identified in LX-2. Variants with significant (FDR < 0.01) allele-differential activity (DAVs) are highlighted in red dots. **(D)** Venn diagram demonstrating the overlapped DAVs in HepG2 and LX-2 cell lines. **(E)** Scatter plots illustrating the allele-differential activities of DAVs between HepG2 and LX-2 cell lines. **(F)** TF motif enrichment analysis of DAVs specific to LX-2 cells, highlighting enrichment of fibrosis-related TFs. **(G)** MPRA-assessed activity of the A and G alleles of rs6726823 in HepG2 and LX-2 cell lines. **(H)** Genomic visualization of the rs6726823 locus near *CASP8*. The position of rs6726823 is highlighted in yellow. Annotations include snATAC chromatin accessibility, liver histone modification profiles, chromatin loops and regulatory links, MPRA-tested regions, and identified DAVs. **(I)** Predicted effect of rs6726823 on IRF binding affinity, based on TF motif disruption analysis. **(J)** MPRA-assessed activity of the A and C alleles of rs13150068 in HepG2 and LX-2 cell lines. **(K)** Genomic visualization of rs13150068 within hepatocyte-specific peak (yellow box) at *HSD17B13* locus with relevant regulatory annotations. **(L)** Predicted effect of rs13150068 on SREBP2 binding affinity, based on TF motif disruption analysis.

The progression of MASLD involves multiple liver cell types, including the activation of hepatic stellate cells. We hypothesized that MASLD risk variants modulate the activity of regulatory elements in a cell type-specific manner. MPRA analysis revealed substantial differences in regulatory element activity between human hepatocyte cells line HepG2 and stellate cell line LX-2, in line with the cell type-specific chromatin accessibility observed (Fig. 3A **and Extended Data Fig. 9A**). Regulatory elements specific to HepG2 cells were enriched for motifs associated with PPARα and ERRγ, key nuclear receptors involved in liver metabolism. In contrast, LX-2-specific elements contained binding sites for fibrosis-related TFs, such as STAT6 (Fig. 3B **and Extended Data Fig. 9B**). These cell type-specific regulatory element activities resulted in more than half of the DAVs exhibiting allele-specific activity exclusively in either HepG2 or LX-2 cells (Fig. 3C–3E **and Extended Data Fig. 9C**). In total, we identified 464 DAVs in LX-2 cells, with the majority being liver eQTLs, located in epigenomic regions, or disrupting specific TF binding motifs (**Extended Data Fig. 9D-9E and Table S3**). Notably, LX-2-specific DAVs disrupted the binding sites of STAT, TEAD, and TCF21 (Fig. 3F **and Extended Data Fig. 9F**), all of which have been implicated in liver fibrosis^39^. For instance, the genetic variant rs6726823 near the *CASP8* gene exhibited significant allele-specific activity in LX-2 but showed far less differential activity in HepG2 (Fig. 3G). Interestingly, the predicted target gene of rs6726823, *CASP8*, has been functionally implicated in hepatic stellate cell homeostasis and liver fibrosis in MASLD mouse models^40,41^. Mechanistically, we found that rs6726823 resides in stellate-specific chromatin peaks and may regulate *CASP8* expression by affecting the binding motif of IRF, a transcription factor family implicated in liver fibrosis (Fig. 3H–3I)^42^. In contrast, the variant rs13150068 displayed allele-specific activity exclusively in HepG2 cells, with no notable differential activity in LX-2 (Fig. 3J). This variant is located in a hepatocyte-specific open chromatin region near *HSD17B13*, which is a liver-enriched gene involved in lipid droplet metabolism and strongly associated with MASLD pathogenesis (Fig. 3K)^43^. Motif analysis suggests that rs13150068 may exert its regulatory effect by disrupting the binding site of SREBP2, a master transcriptional regulator of lipid biosynthesis (Fig. 3L). These findings underscore the importance of selecting disease-relevant cell lines to accurately identify bona fide functional variants.

### Context-dependent activity of DAVs

**Figure 4:**
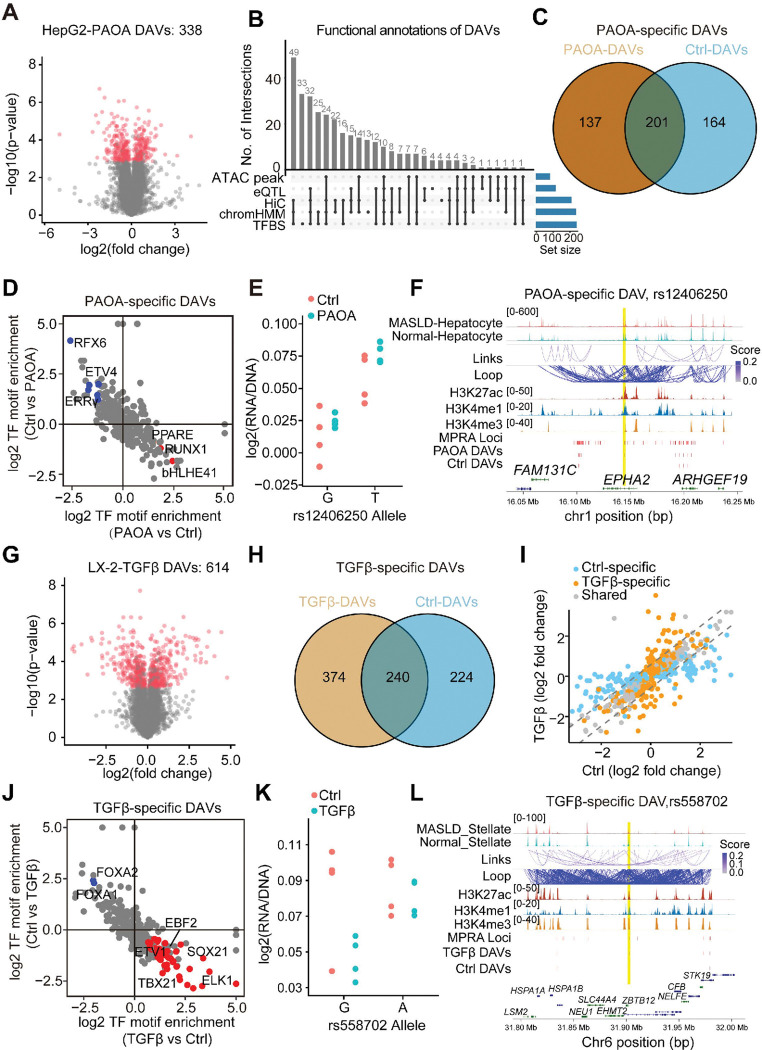
Context-dependent activity of DAVs **(A)** Volcano plot showing the effect sizes and *p* values of MPRA variants identified in PAOA-treated HepG2 cell. Variants with significant (FDR < 0.01) allele-differential activity (DAVs) are highlighted in red dots. **(B)** Intersection sizes of PAOA-HepG2 DAVs overlapping with functional annotations such as ATAC peaks from snATAC-seq data, liver eQTLs, liver chromatin interactions, transcription factor binding motifs disruption, and ChromHMM states. **(C)** Venn diagram demonstrating the overlapped DAVs identified in Ctrl- and PAOA-treated HepG2. **(D)** TF motif enrichment analysis of stimuli-specific DAVs. Red and blue colors denote significantly enriched motifs (q < 0.1). The PPARE motif is highlighted in light salmon to indicate nominal significance (p <0.01). **(E)** MPRA-assessed activity of the T and G alleles of rs12406250 in Ctrl- and PAOA-treated HepG2. **(F)** Genomic visualization of rs12406250 within MASLD-enhanced hepatocyte peak (yellow box) at *EPHA2* locus. The position of rs12406250 is highlighted in yellow. Annotations include snATAC chromatin accessibility, liver histone modification profiles, chromatin loops and regulatory links, MPRA-tested regions, and identified DAVs. **(G**)Volcano plot showing the effect sizes and *p* values of MPRA variants identified in TGFb-treated LX-2 cell. Variants with significant (FDR < 0.01) allele-differential activity (DAVs) are highlighted in red dots. **(H)** Venn diagram demonstrating the overlapped DAVs in Ctrl- and TGFb-treated LX-2. **(I)** Scatter plots illustrating the allele-differential activities of MPRA variants between TGFb- and Ctrl-treated conditions. **(J)** TF motif enrichment analysis of TGFb stimuli-specific DAVs. Colors indicate significantly enriched motifs (q < 0.1). **(K)** MPRA-assessed activity of the A and G alleles of rs558702 in Ctrl- and TGFb-treated LX-2. **(L)** Genomic visualization of rs558702 (yellow box) at *ZBTB12*locus with relevant regulatory annotations.

Since the progression of MASLD is shaped by the complex interplay between genetic predisposition and environmental factors^2^, we further delineated the functional variants under various environmental stimuli, including oleic acid and palmitic acid (PAOA), and TGFβ to mimic the metabolic stress in MASLD livers^44^. Hundreds of regulatory elements were induced in HepG2 under PAOA condition, with PAOA-induced elements being enriched for the binding motifs of metabolic nuclear receptors, ERRα and TRβ (**Extended Data Fig. 10A-10C**). A total of 338 DAVs were identified in PAOA-treated hepatocytes, with most of them having at least two functional annotations (Fig. 4A–4B **and Extended Data Fig. 10D-10E and Table S4**). Notably, one-third of the DAVs were stimulus-dependent, displaying variable allele-specific activity between control and PAOA conditions (Fig. 4C **and Extended Data Fig. 10F**). Of note, these PAOA-specific DAVs appeared to preferentially affect the PPARE and RUNX1 motifs (Fig. 4D). In particular, rs12406250 exhibited higher allele-specific activity under PAOA conditions compared to control (Fig. 4E). This variant is located in an intronic open chromatin peak of the *EPHA2* gene, whose expression and signaling are known to be altered during MASLD pathogenesis (Fig. 4F)^45^. Moreover, the intensity of the peak containing rs12406250 was elevated in MASLD-hepatocytes relative to normal hepatocytes (Fig. 4F), suggesting the role of rs12406250 in regulating the chromatin accessibility in MASLD.

We further identified 614 DAVs in LX-2 cells in response to TGFβ treatment, and these DAVs likely exert their regulatory effects by modulating chromatin accessibility and TF binding dynamics (Fig. 4G **and Extended Data Fig. 10G-10I and Table S5**). Notably, most of these DAVs exhibited differential allele-specific activity compared to the basal state, suggesting that TGFβ alters the genomic binding landscape and reveals context-specific variant function (Fig. 4H–4J). For example, rs558702, a DAV located within a stellate cell-specific open chromatin region, exhibited enhancer activity exclusively upon TGFβ treatment, highlighting its context-specific regulatory role (Fig. 4K–4L). Together, these findings underscore how environmental cues such as PAOA and TGFβ exposure can unmask the functional consequences of noncoding variants and provide mechanistic insight into gene-environment interactions driving MASLD pathogenesis.

### Disease-associated transcriptional programs driven by DAVs

**Figure 5:**
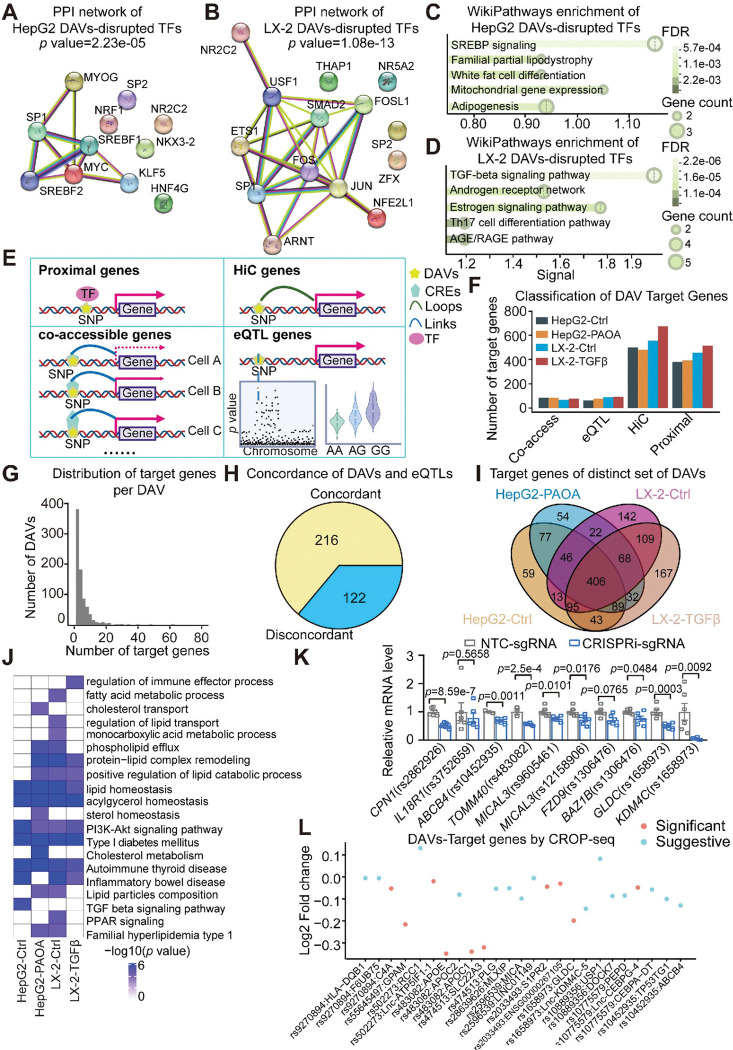
Determination of target genes associated with MASLD DAVs. **(A-B)** Protein-protein interaction (PPI) networks of TFs significantly disrupted by DAVs in HepG2 (A) and LX-2 (B) cells, based on STRING database analysis. Edge thickness reflects confidence scores of interactions. **(C-D)** Wikipathways enrichment analysis of TFs significantly perturbed by DAVs in HepG2 (C) and LX-2 (D) cells. **(E)** Schematic diagram illustrating four approaches for assigning target genes to DAVs, including proximal genes, genes identified through Hi-C chromatin interactions, co-accessible genes, and eQTL-associated genes. **(F)** Comparison of target gene numbers identified by the four approaches in various DAV sets. **(G)** Distribution of the number of target genes assigned to each DAV through four integrated methodologies. **(H)** Pie chart illustrating the number of DAVs overlapping with liver eQTLs that exhibit concordant or discordant allele-specific MPRA activity relative to eQTL effect sizes. **(I**)Venn diagram showing the overlap of target genes assigned by different DAV subsets. **(J)** Enriched biological pathways associated with target genes from multiple DAV groups. **(K)** mRNA expression of nominated target genes in HepG2 cell following introduction of CRISPRi machinery (n = 6). Data are shown as mean ± SEM (Student’s t test). **(L)** Fold change in expression of nominated target genes for candidate sgRNAs identified by single-cell CROP-seq. (Significant, q < 0.1, red; Suggestive, *p* < 0.05, blue).

To elucidate the transcriptional networks regulated by context-specific DAVs, we systematically analyzed the TFs disrupted by DAVs in HepG2 and LX-2 cells. Protein-protein interaction network analysis revealed that TFs perturbed by HepG2-DAVs formed a tightly connected transcriptional module centered around SREBP signaling and lipid metabolism, whereas LX-2-perturbed TFs were enriched in a network linked to TGFβ signaling (Fig. 5A–5D **and Extended Data Fig. 11A-11B**). These results indicate that MASLD-associated DAVs converge on distinct, cell-type-specific transcriptional programs, with TFs functioning cooperatively within coordinated, disease-relevant regulatory circuits.

To accurately identify the target genes of these DAVs, we integrated multiple orthogonal datasets, including public liver eQTL database, chromatin interaction data, and co-accessibility analyses from snATAC, as well as proximal gene information (Fig. 5E). This comprehensive approach enabled us to assign between 794 and 1,009 target genes for different set of DAVs, with 995 DAVs having at least one associated target gene (Fig. 5F–5G **and Extended Data Fig. 12A-12F**). Among these, 338 DAVs overlapped with known liver eQTLs, and notably, 64% exhibited concordant effect directions between reporter activity and eQTL effect size (Fig. 5H **and Extended Data Fig. 12G-12J**). The remaining 36% discordance likely reflects the cellular heterogeneity of liver tissue and the context-dependent nature of chromatin regulation. Interestingly, gene ontology analysis of these potential target genes revealed many biological processes associated with MASLD progression, including lipid metabolism, immune responses, and the TGFβ signaling pathway (Fig. 5I–5J). Moreover, transcriptional regulatory network analysis revealed that many DAV target genes are regulated by key MASLD-related TFs, including SREBF1, MLXIPL (ChREBP), ERG, and TCF7L2 (**Extended Data Fig. 12K**).

To validate the functional predictions, we deactivated the genomic regions spanning 8 randomly selected DAVs using CRIPSR interference (CRISPRi) approach and examined the expression levels of the nominated target genes. Over 90% of the potential target genes showed reduced expression levels, consistent with predictions (Fig. 5K **and Extended Data Fig. 13A**). Conversely, when CRIPSR activation (CRISPRa) machinery was introduced into the cells, most target genes exhibited increased expression levels (**Extended Data Fig. 13A-13B**). To systematically identify the target genes of MASLD-associated DAVs, we employed a CROP-seq approach that integrates scRNA-seq with CRISPR screening^46^. A lentiviral-based CROP-seq library was generated with high complexity and uniformity, comprising 40 sgRNAs targeting 20 genomic regions spanning MASLD-associated DAVs with strong allelic effects and well-annotated metabolic target genes (**Extended Data Fig. 13C and Table S6**). scRNA-seq of CROP library-infected HepG2 cells revealed 67,700 high-quality cells and identified 18 distinct cell clusters (**Extended Data Fig. 13D-13F**). We then associated the DAVs with the previously nominated target genes and identified 25 unique gene-sgRNA pairs, where the expression levels of the target genes were significantly lower in DAVs sgRNA-infected cells compared to all other sgRNA-infected cells (Fig. 5L). This analysis offers a robust strategy for narrowing down the pool of target genes associated with functional variants, providing mechanistic insights into MASLD progression.

### Functional characterization of DAVs modulating APOA5, ANGPTL3, and LPL in triglyceride metabolism

**Figure 6:**
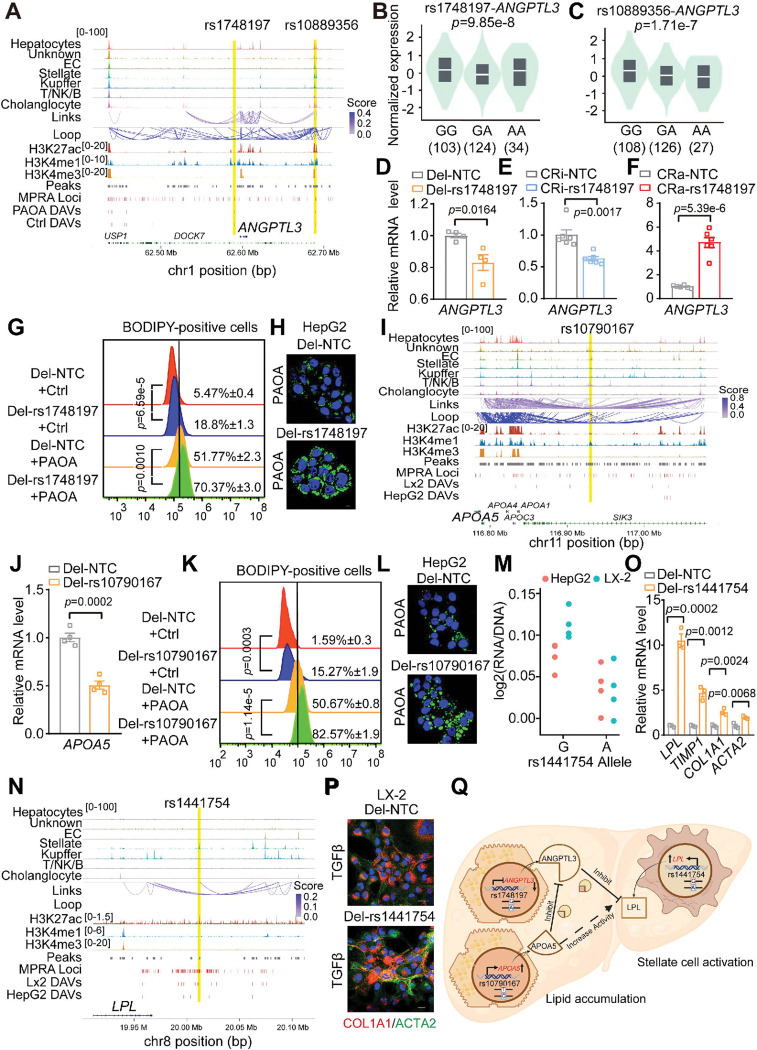
Functional characterization of DAVs modulating APOA5, ANGPTL3, and LPL in triglyceride metabolism. **(A)** Genomic visualization of rs1748197 and rs10889356 (highlighted in yellow) at the *ANGPTL3* locus, with annotations including chromatin accessibility, regulatory elements, and DAV signals. **(B-C)** Expression levels of *ANGPTL3* in human liver samples across different genotypes of rs10889356 and rs1748197 from GTEX database. **(D-F)** Relative mRNA levels of *ANGPTL3*in HepG2 cells following CRISPR-mediated deletion (Del) (D), CRISPR interference (CRISPRi, CRi) knockdown (E), or CRISPR activation (CRISPRa, CRa) (F). Data represent mean ± SEM from 4–6 biological replicates. **(G)** Flow cytometry analysis of lipid accumulation in rs1748197-deleted HepG2 cells treated with or without PAOA for 24 h (n = 3). **(H)** Representative images of BODIPY staining in rs1748197-deleted HepG2 treated with PAOA for 24 h. Scale bar: 10 μm. **(I)** Genomic view of rs10790167 (yellow highlight) at the *APOA5* locus with relevant regulatory annotations. **(J)** mRNA expression of *APOA5* in rs10790167-deleted HepG2. Data represent mean ± SEM from four biological replicates. **(K)** Flow cytometry analysis of lipid content in rs10790167-deleted HepG2 cells following 24 h PAOA treatment (n = 3). **(L)** Representative images of BODIPY staining in rs10790167-deleted HepG2 treated with PAOA for 24h. Scale bar: 10 μm. **(M)** MPRA-based regulatory activity of the G and A alleles of rs1441754 in HepG2 and LX-2 cell lines, showing allele-specific enhancer function. **(N)** Genomic visualization of rs1441754 (yellow box) at the *LPL* locus, annotated with regulatory features and DAV assignment. **(O)** mRNA expression of *LPL* and fibrosis associated genes, (*TIMP1*, *COL1A1* and *ACTA2*) in rs1441754-deleted LX-2. Data represent mean ± SEM from three biological replicates. **(P)** Representative immunofluorescence images of COL1A1 and ACTA2 in rs1441754-deleted LX-2 cells after 48-hour TGFβ stimulation. Scale bar: 25 μm. **(Q)** Schematic summary model illustrating how DAVs at the *ANGPTL3*, *APOA5*, and *LPL* loci coordinate to regulate triglyceride metabolism and fibrogenic responses in hepatocytes and hepatic stellate cells. Data are shown as mean ± SEM (Student’s t test).

With the identification of potential functional DAVs and their annotated target genes, we next investigated how these DAVs influence lipid metabolism, a key aspect of MASLD, in the liver. APOA5, ANGPTL3/8, and LPL form a tightly regulated network that plays a central role in triglyceride metabolism and systemic lipid homeostasis^47^. We then focused on two DAVs (rs1748197 and rs10889356) that are near the *ANGPTL3* locus (Fig. 6A), a well-known inhibitor of LPL activity^48^, and have been previously identified as risk variants associated with MASLD ^7^. Although neutralizing antibodies targeting ANGPTL3 have been approved to reduce lipoprotein levels in individuals with homozygous familial hypercholesterolemia, the inhibition of hepatic ANGPTL3 using antisense oligonucleotide (ASO) drug Vupanorsen unexpectedly led to liver lipid accumulation, ultimately resulting in the termination of the clinical trial^49,50^. We discovered that DAV rs1748197 is located in the promoter region of the *ANGPTL3* gene, and confirmed that rs1748197 acts as an eQTL variant for *ANGPTL3* (Fig. 6A–6B). Deletion of a small genomic region containing rs1748197 or deactivation of this genomic region using CRISPRi reduced the expression of *ANGPTL3*, while genomic activation using CRISPRa enhanced the expression of *ANGPTL3* (Fig. 6D–6F **and Extended Data Fig. 14A**). Mechanistically, rs1748197 is predicted to alter the binding motif of MYC, a transcription factor implicated in the pathogenesis of alcoholic liver disease (**Extended Data Fig. 14B**)^51^.

Meanwhile, another DAV rs10889356 resides in an enhancer region that interacts with the *ANGPTL3* promoter (Fig. 6A). Public liver eQTL data reveals a strong association between rs10889356 and *ANGPTL3* expression, with livers carrying G allele having higher *ANGPTL3* expression (Fig. 6C). Indeed, inhibition or activation of this enhancer region reduced or increased *ANGPTL3* expression, respectively (**Extended Data Fig. 14C**). Motif analysis further revealed that rs10889356 perturbs a GABPA binding motif, and reporter assays confirmed that the G allele promotes enhancer activity, likely through increased GABPA recruitment (**Extended Data Fig. 14D-14E**). Functionally, deletion of the small genomic regions containing rs1748197 and rs10889356 individually in HepG2 cells resulted in greater lipid accumulation compared to wild-type HepG2 cells under both basal and PAOA-treated conditions (Fig. 6G–6H **and Extended Data Fig. 14F-14G**). These findings were further confirmed in these DAVs-deleted hepatocyte spheroids treated with PAOA (**Extended Data Fig. 14H**). These data suggest that MASLD-associated DAVs rs1748197 and rs10889356 modulate the expression of *ANGPTL3*, which negatively regulates hepatic lipid metabolism.

We further identified rs10790167 as a HepG2-specific DAV with robust allele-specific activity and no significant regulatory effect in LX-2 cells (Fig. 6I). This variant is located in a hepatocyte-specific open chromatin region and establishes an enhancer-promoter (E-P) interaction with the promoter of *APOA5*, which is a known activator of LPL activity and closely linked to MASLD progression^52^. Motif analysis revealed that rs10790167 disrupted the binding motifs of several nuclear receptors, including PPAR and RXR (**Extended Data Fig. 14I**). Deletion of rs10790167 significantly reduced the expression of *APOA5* and led to marked accumulation of lipid droplets in HepG2 cells, suggesting a causal role for this variant in dysregulated lipid metabolism (Fig. 6J–6L).

In contrast, the genetic variant rs1441754 near the lipoprotein lipase (*LPL*) gene, previously identified as a cholesterol-associated variant, exhibited significant allele-specific activity in LX-2 but showed far less differential activity in HepG2 (Fig. 6M–6N)^53^. Luciferase reporter assays confirmed this cell type-specific enhancer activity, with significant allele-specific effects observed exclusively in LX-2 cells, but not in HepG2 or Huh7 hepatocyte cell lines (**Extended Data Fig. 14J**). Interestingly, the predicted target gene of rs1441754, *LPL*, is upregulated in hepatic stellate cells and exacerbates liver fibrosis in MASLD in mouse models^54^. Mechanistically, we found that rs1441754 resides in stellate-specific chromatin peaks and may regulate *LPL* expression by affecting the binding motif of RUNX (**Extended Data Fig. 14K**). Strikingly, deletion of the rs1441754-containing region in LX-2 resulted in a significant increase in *LPL* expression, suggesting its role as a negative regulatory element in stellate cells (Fig. 6O). Functionally, rs1441754 deletion also resulted in the upregulation of multiple fibrosis-associated genes in LX-2 cells (Fig. 6O–6P). Together, these findings highlight the integrative roles of context-specific DAVs in modulating key regulators of triglyceride metabolism including APOA5, ANGPTL3, and LPL, and their downstream contributions to altered lipid homeostasis and hepatic stellate cell activation in MASLD (Fig. 6Q).

In addition to the role of rs1441754 in stellate cell activation, we identified another regulatory variant, rs66477683, which modulates *MERTK* expression (**Extended Data Fig. 15A-15B**). Given prior reports linking MERTK to liver fibrosis^55^, our findings suggest that rs66477683 may represent a fibrosis-driving variant acting through transcriptional control of *MERTK* (**Extended Data Fig. 15C-15D**).

### Functional linking of rs474513 to SLC22A3 and hepatic lipid homeostasis

**Figure 7:**
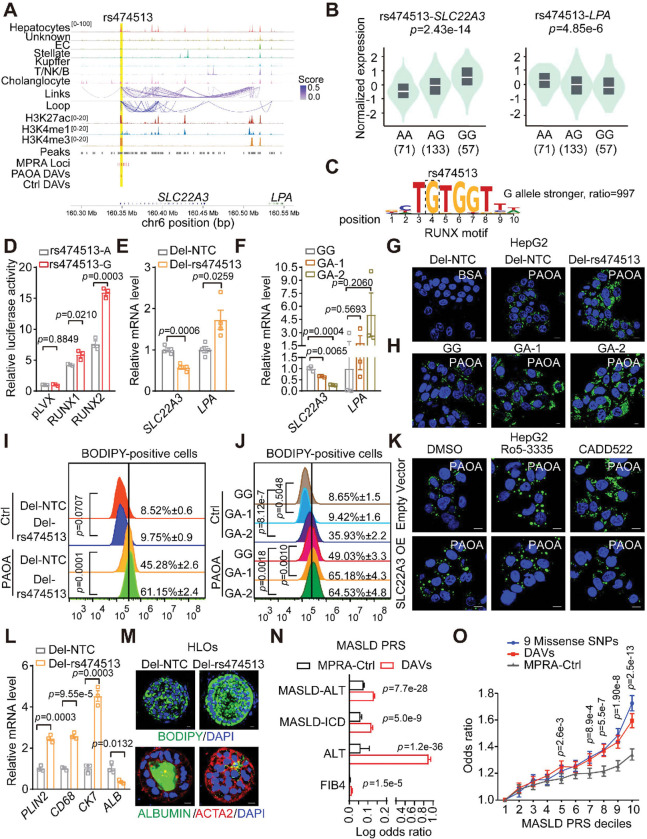
DAV rs474513 controls *SLC22A3* gene expression and lipid metabolism. **(A)** Genomic visualization of rs474513 (highlighted in yellow) located within a hepatocyte-specific accessible chromatin peak at the *SLC22A3* locus, with annotations including chromatin accessibility, regulatory elements, and DAV signals. **(B)** Expression levels of *SLC22A3*and *LPA* in human liver samples across different genotypes of rs474513 from GTEX database. **(C)** Predicted disruption of RUNX transcription factor binding by rs474513, based on TF motif analysis. **(D)** Luciferase reporter assay showing allele-specific regulatory activity of rs474513 in 293T cells transfected with control, RUNX1, or RUNX2 expression plasmids (n = 3). **(E)** mRNA expression of *SLC22A3* and *LPA* in HepG2 cell following deletion of a small genomic region containing rs474513 (n = 4). **(F)** mRNA expression of *SLC22A3* in WT and G>A heterozygous HepG2 cell (n = 3). **(G-H)** Representative BODIPY staining images showing lipid droplet accumulation in rs474513-deleted (G) and rs474513 G>A gene-edited (H) HepG2 cells following 24-hour treatment with PAOA. Scale bars, 10 μm. **(I-J)** Flow cytometry analysis of lipid content in HepG2 cells with rs474513 deletion (I) or G>A editing (J), with or without PAOA treatment for 24 h (n = 4). **(K)** Effect of RUNX inhibition on SLC22A3-mediated lipid accumulation in HepG2 cells. Representative BODIPY staining images following treatment with RO5–3335 (50 μM) or CADD522 (50 μM). Scale bars, 10 μm. **(L)** Expression of marker genes for various liver cell types in HLOs derived from hiPSCs treated with or without PAOA for 4 days (n = 3). **(M)** Representative images of BODIPY staining and immunofluorescence in HLOs. Top panel: BODIPY staining (green) showing lipid accumulation. Bottom panel: ALBUMIN (green) and ACTA2 (red) immunostaining. Scale bars, 10 μm. (**N)** Odds ratios of polygenic risk scores (PRS) constructed from 114 LD-independent DAVs and matched MPRA control variants for MASLD-related traits in the Million Veteran Program (MVP). Variant weights were derived from effect sizes reported in the GWAS by Miao et al. as shown in Table S7. PRS were evaluated for four phenotypes: (1) MASLD-ALT, defined as ALT > 40 U/L in men or > 30 U/L in women; (2) MASLD-ICD, based on diagnostic ICD codes; (3) continuous ALT levels; and (4) fibrosis progression, assessed by the FIB-4 index. PRS were calculated in a cohort of 90,408 chronic ALT elevation (cALT) cases and 128,187 controls. Odds ratios represent the effect size per standard deviation increase in PRS. (**O)** Odds ratios for each decile of PRSs constructed from 9 missense SNPs, DAVs, and MPRA-Ctrl variants, compared with their respective bottom deciles. For the top 4 deciles, the DAVs PRS demonstrated prediction power similar to that of the PRS constructed from 9 missense SNPs, with both outperforming the PRS constructed from MPRA-Ctrl in predicting MASLD-ALT. Data are presented as mean ± SEM, log odds ratios (log ORs), or odds ratios (ORs) with 95% confidence intervals (CIs), as indicated. Statistical significance was assessed using two-tailed Student’s t-test.

Notably, DAV rs474513, previously linked to obesity and type 2 diabetes^56^, is located in hepatocyte-specific open chromatin regions near the promoter of the *SLC22A3* gene. Chromatin interaction data also revealed a strong enhancer-promoter interaction between the genomic region containing rs474513 and the *LPA* gene, suggesting the regulatory potential of rs474513 on *SLC22A3* and *LPA*(Fig. 7A). Interestingly, public liver eQTL data showed that the genotype of rs474513 is closely associated with the expression levels of both *SLC22A3* and *LPA*, but in opposite directions (Fig. 7B). Livers carrying the alternative allele G exhibited higher expression of *SLC22A3* and lower expression of *LPA* compared to those carrying the reference allele A. Mechanistically, rs474513 modulates the binding motif for RUNX, a transcription factor known to be involved in MASLD progression^57^. The agreement score with the RUNX consensus motif was 997-fold greater for the G allele than for the A allele (Fig. 7C). Consistently, overexpression of RUNX1/2 increased the activity of reporters carrying the G allele compared to those with the A allele (Fig. 7D). In contrast, inhibition of RUNX2 activity using small molecule CADD522 reduced *SLC22A3* expression, but had no effect in HepG2 cell where a small genomic region containing rs474513 was deleted (**Extended Data Fig. 16A-16B**). Moreover, deletion of the genomic region containing rs474513 led to decreased *SLC22A3* expression and increased *LPA* expression, in line with the eQTL data (Fig. 7E). Similar gene expression patterns were observed when the genomic region was inactivated using CRISPRi technology (**Extended Data Fig. 16C**). Importantly, the G > A-edited heterozygous HepG2 cells also exhibited decreased *SLC22A3* expression (Fig. 7F **and Extended Data Fig. 16D**).

To examine the role of rs474513 in lipid metabolism associated with MASLD, we treated the rs474513-deleted or gene-edited HepG2 with PAOA to model steatosis. Interestingly, we found that lipid accumulation was increased in rs474513-deleted and G > A-edited HepG2 cells treated with PAOA (Fig. 7G–7J). Given the established role of RUNX in mediating the transcriptional activity of rs474513, we next treated HepG2 with the RUNX1/2 inhibitors Ro5–3335 and CADD522. Pharmacologic inhibition of RUNX signaling led to reduced *SLC22A3* expression and further exacerbated lipid accumulation, mirroring the effects of rs474513 deletion (Fig. 7K **and Extended Data Fig. 16E-16F**). Importantly, ectopic overexpression of SLC22A3 rescued lipid accumulation phenotype (Fig. 7K **and Extended Data Fig. 16E-16F**), confirming that SLC22A3 acts downstream of the rs474513-RUNX regulatory axis to maintain hepatic lipid homeostasis.

To further investigate the physiological function of rs474513 in MASLD-relevant organoid model^58^, we deleted a small genomic region containing rs474513 in human induced pluripotent stem cell (hiPSC) line and observed a reduction in *SLC22A3* expression (**Extended Data Fig. 16G-16H**), consistent with the above finding. Importantly, the deletion of rs474513 did not impair the efficiency of hepatic differentiation in the hiPSC line (**Extended Data Fig. 16I**). We then differentiated both the wild-type and rs474513-depleted hiPSC lines into human liver organoids (HLOs) and exposed them to free fatty acids to model the steatosis process^58^. Interestingly, the rs474513-depleted HLOs exhibited higher lipid accumulation compared to wild-type HLOs, along with increased expression of *PLIN2*, a gene involved in lipid storage and metabolism (Fig. 7L–7M). We also observed elevated levels of *CD68* and *CK7* in rs474513-depleted HLOs (Fig. 7L–7M), indicating an enhanced inflammatory response and ductal reaction. Furthermore, the higher ACTA2 and lower ALBUMIN expression implied the potential risk of fibrosis.

### Functional DAVs improve MASLD risk prediction

Polygenic risk scores (PRS) aggregate the effects of multiple genetic variants to estimate an individual’s likelihood of developing a disease^59^. To assess whether hepatic DAVs can effectively predict MASLD risk, we compared the performance of the PRS constructed from 114 LD-independent MPRA-prioritized variants (DAVs) **(Table S7**) to the control PRS (MPRA-Ctrl) constructed from randomly selected, matched set of MPRA total variants using data from the Million Veteran Program (MVP)^60^. Our analysis revealed that hepatic DAVs PRS outperformed the control PRS in predicting various MASLD-related traits, including MASLD defined by chronic elevation of liver enzyme alanine aminotransferase (MASLD-ALT), MASLD defined by international classification of disease (ICD) codes (MASLD.ICD), ALT levels, and fibrosis index FIB4 progression (Fig. 7N). Notably, the individuals with DAV PRS scores in the top 10% exhibited a 1.6-fold higher risk of developing MASLD compared to the ones in the lowest 10%, which is better than the performance of MPRA-Ctrl PRS, and comparable to the PRS built from nine well-established missense variants contributing to MASLD (PRS9), including the rs738409 (I148M) variant of the gene *PNPLA3* and rs58542926 (E167K) of the gene *TM6SF2* (Fig. 7O **and Table S8**)^4^. We further benchmarked the DAV-PRS in the *All of Us Research Program* against previously published MASLD PRS models and found that, while our MPRA-prioritized PRS performed similarly to PRS9, its predictive power was modestly lower than PGS002732, the best-performing published model (**Extended Data Fig. 16J**)^4^. These data underscore the power of our approach in identifying functional variants linked to MASLD, which can also be extended to other complex diseases.

## Discussion

Fine-mapping causal variants for diseases or complex traits continues to be a major hurdle in the field of genetics^61^. Numerous computational algorithms have been developed, employing various statistical and computational strategies to fine-map genetic variants. For example, FINEMAP and DAP utilize Bayesian frameworks to estimate posterior probabilities of causality; PAINTOR and fGWAS incorporate functional annotations to prioritize variants; Coloc and eCAVIAR identify shared causal variants between complex traits^62^. Additionally, deltaSVM enhances fine-mapping by providing functional predictions for non-coding variants that disrupt transcription factor binding using machine learning techniques^63^. However, computational prediction algorithms for prioritizing functional variants are often not concordant among themselves and are frequently inconsistent with experimental validation^9^. Here, by integration of snATAC, high-throughput MPRA, and CROP-seq data, we identified functional causal variants and revealed key disease-associated cell types or cell states and elucidated the transcriptional programs influenced by these functional variants. Remarkably, functional variants identified in this way regulate hepatic lipid metabolism and stellate cells activation and effectively predict MASLD risk.

To our knowledge, this is the first study to comprehensively profile the heterogeneity of chromatin landscapes in individuals at different stages of MASLD and thus provides a high-quality human liver regulome resource. Integrating GWAS variants with single-cell chromatin landscape data revealed that MASLD risk variants are enriched in disease-specific hepatocyte and stellate cell states. Moreover, by combining motif activity and risk variant enrichment analyses at the single-cell level, we demonstrated how these MASLD risk variants regulate gene expression. Furthermore, the single-cell chromatin accessibility data enabled us to nominate target genes of risk variants using co-accessibility analysis. Notably, snATAC-seq studies using large cohorts of MASLD liver samples will enhance the statistical power to pinpoint the functional variants by performing chromatin accessibility QTL (caQTL) or allele-imbalance analyses^64^.

Importantly, our MPRAs were conducted using two disease-relevant cell lines under MASLD-associated environmental conditions, demonstrating that these DAVs exhibit cell type-specific and stimulus-dependent activity. This highlights the importance of selecting appropriate cell lines and cellular contexts for identifying functional SNPs. Notably, only 64% of DAVs exhibited concordant effect directions between reporter activity and eQTL effect size, while the remaining 36% showed discordance, likely reflecting the cellular heterogeneity of liver tissue and the context-dependent nature of chromatin environments. Future implementation of this approach in human liver organoids or *in vivo* models may provide more physiologically relevant insights^14^. Moreover, using various CRISPR approaches, we identified MASLD-associated transcriptional programs. We observed that rs474513 affects the expression of *SLC22A3* and *LPA* in opposite directions, demonstrating how a single variant can disparately regulate multiple genes^65^. Conversely, both rs1748197 and rs10889356 regulate the expression of *ANGPTL3*, suggesting synergistic effects of these genetic variants. Remarkably, we identified several MASLD-associated DAVs that regulate the core regulatory network of LPL activity, including APOA5 (an LPL activator), ANGPTL3 (an LPL inhibitor), and LPL itself. These findings suggest that MASLD development is possibly mediated by local paracrine effects on LPL activity in the liver. Importantly, using physiologically relevant human liver organoids and gene-editing tools, we demonstrated the causal roles of these genetic variants in lipid metabolism associated with MASLD.

In sum, our study combined high-throughput MPRA, snATAC-seq, and CROP-seq to identify functional causal variants and their target genes in MASLD-related cell types. This broadens our understanding of genetic mechanisms underlying MASLD risk, and will be a valuable guide to prediction and treatment of MASLD and related disorders. Moreover, this approach may be employed to better understand other complex human diseases.

## Methods

### Cell lines and culture

Human hepatoma cell line HepG2 and Huh-7, human hepatic stellate cell line LX-2, and human embryonic kidney (HEK) 293T cell line were cultured in Dulbecco’s Modified Eagle’s Medium (DMEM) supplemented with 10% fetal bovine serum (FBS). Cells were maintained in a humidified incubator at 37 °C with 5% CO_2_and supplemented with 100 U/mL penicillin and 100 μg/mL streptomycin. To establish an *in vitro* lipotoxic model, HepG2 or Huh-7 cells were treated with 0.15 mM palmitic acid (PA) and 0.3 mM oleic acid (OA). Both fatty acids were dissolved in 0.5% fatty acid-free bovine serum albumin (BSA). The treatment was administered for 24 h. To establish an *in vitro* fibrosis model, LX-2 cells were treated with 20 ng/mL transforming growth factor-beta 1 (TGFβ_1_) for 48 h.

### Human iPSCs culture

Human induced pluripotent stem cell (iPSC) lines were generated following the study protocol approved by The University of Pennsylvania Human Subjects Research Institutional Review Board, as previously reported^66^. iPSCs were cultured in mTeSR^™^ medium at 37 °C with 5% CO_2_. Gene-edited stable monoclonal iPSC lines were produced as previously described^67^. In brief, human iPSCs were infected with Exci-sgRNA lentivirus co-expressing Cas9 and selected for 3 days using puromycin (0.5 μg/ml) and blasticidin (5 μg/ml). The surviving iPSCs were then subcloned at low seeding density and cultured for one week. Following colony isolation and expansion, Sanger sequencing was performed to identify stable DAV-deleted monoclonal human iPSC lines.

### Human studies

Normal and MASLD human livers for snATAC-seq were provided by the Liver Tissue Cell Distribution System, Minneapolis, Minnesota, which was funded by National Institutes of Health contract no. HSN276201200017C. Additional human livers were obtained from SEKISUI XenoTech. All studies using human samples were exempted by the University of Pennsylvania Institutional Review Board.

### HepG2 CRISPRi/a cell lines construction

The CRISPRi_HepG2 cell line was generated by targeted integration of a UCOE-CAG promoter-driven dCas9-KRAB/MeCP2-T2A-BSD expression cassette into the AAVS1 “safe harbor” site using CRISPR/Cas9. Donor plasmid (pUC19-AAVS1-dCas9-KRAB/MeCP2) and AAVS1 targeting plasmid (PX330-sgAAVS1_human) were co-transfected into HepG2 cells using Lipofectamine 3000 reagent. Blasticidin selection (30 μg/mL) commenced 72 h post-transfection and was maintained for at least 7 days. Expression of dCas9-KRAB/MeCP2 was evaluated by immunofluorescence.

The CRISPRa_HepG2 cell line was established by infecting HepG2 cells with lentiviruses containing a reverse tetracycline-controlled trans-activator (rtTA) expression cassette co-expressing blasticidin S deaminase (BSD) and a 3G-Tet-regulated dCas9-VPR expression cassette. Blasticidin selection (30 μg/mL) was initiated 72 h post-infection and maintained for at least 7 days. Expression of dCas9-VPR was induced by treating cells with doxycycline (5 μg/mL) for 3 days and subsequently evaluated by immunofluorescence.

### Plasmids construction

The donor plasmid (pUC19-AAVS1-dCas9-KRAB/MeCP2) was generated by cloning a UCOE-CAG-dCas9-KRAB/MeCP2-T2A-BSD expression cassette alongside with AAVS1 homology arm (HA) into pUC19 backbone. The HA-L, HA-R and UCOE element were amplified from human genome. The CAG promoter was amplified from SMARTvector. The dCas9-KRAB/MeCP2, T2A-BSD-WPRE and SV40-polyA fragments were amplified from the pLX-TRE-dCas9-KRAB/MeCP2-BSD vector. For PX330-sgAAVS1 plasmid, annealed oligo duplex (sgAAVS1_human) was prepared and cloned into BbsI cut pX330-U6-hSpCas9 backbone.

The TetON-dCas9-VPR-BSD plasmid was generated by replacing the KRAB/MeCP2 expression sequence in the pLX-TRE-dCas9-KRAB/MeCP2-BSD vector with VPR expression sequence amplified from SP-dCas9-VPR plasmid. All the primers are shown in Supplemental Table S9. To produce lentiviruses, the lentivirus expression vector and the packaging plasmid mix (psPAX2 and pMD2.G) were co-transfected into HEK-293T cells. The lentivirus supernatant was harvested and stored in aliquots at −80°C until use.

For pcDNA3.1-*SLC22A3* overexpression (OE) plasmid construction. The human *SLC22A3* CDS gene was obtained using HepG2 cDNA as a template by PCR amplification. The expression plasmid vector (pcDNA3.1+) was linearized by NheI and KpnI (NEB, Beijing, China) restriction enzyme digestion. The pcDNA3.1-*SLC22A3* vector was constructed according to ClonExpress Ultra One Step Cloning Kit.

### Liver processing and nuclei isolation

Isolation of nuclei for snATAC-seq was performed as previously described ^17^. Briefly, frozen human liver tissue was homogenized using a Dounce homogenizer in lysis dilution buffer containing 10 mM Tris-HCl (pH 7.5), 10 mM NaCl, 3 mM MgCl_2_, 1% BSA, and cOmplete^™^ Protease Inhibitor Cocktail. The homogenate was centrifuged, and the pellet was resuspended in lysis dilution buffer. To the nuclei suspension, lysis buffer (10 mM Tris-HCl, pH 7.5; 10 mM NaCl; 3 mM MgCl_2_; 1% BSA; 0.1% Tween-20; 0.1% NP-40; 0.01% digitonin; cOmplete^™^ Protease Inhibitor Cocktail) was added. The nuclei were washed and resuspended in lysis dilution buffer. Nuclei were further purified using the Nuclei Isolation Kit with a 1.8 M sucrose cushion, following the manufacturer’s instructions. After centrifugation at 30,000 g for 45 minutes at 4 °C, the nuclei pellet was collected and resuspended in PBS containing 1% BSA. The nuclei were filtered through a 40 μm cell strainer before loading onto the 10X Genomics Chromium Controller.

### snATAC-seq libraries construction and sequencing

snATAC-seq libraries were prepared according to the manufacturer’s instructions of 10XGenomics (PN-1000176). Libraries were sequenced with Novogene (https://www.novogene.com/us-en/). 400 million paired-end reads were obtained for each sample using the sequencing parameters recommended in 10X Genomics guide.

### Cluster analysis of snATAC

snATAC-seq data were preprocessed using the 10X Genomics pipeline (Cell Ranger ATAC v2.1.0) aligned to the hg38 genome assembly with default parameters. Samples from different patients were processed separately. High-quality individual cells were retained for downstream analysis (1,000 < unique barcode number < 6,000; reads in peaks percent > 15%; blacklist ratio < 0.05; TSS enrichment > 3; nucleosome signal < 4). Quality control measurements and filtering were conducted using Signac (v. 1.12.9003)^68^. After quality control, all samples were merged into a single Seurat object. The integration function in Signac was then employed to remove batch effects. Unsupervised clustering was performed at a resolution of 0.4. Cell types were identified by integrating liver snRNA data with groups determined through unsupervised clustering. Motif activity was assessed using chromVAR (v. 1.24.0)^69^, and pseudotime scores were calculated with Monocle3 (v. 1.3.4). The pseudotime analysis was further confirmed using StaVia (v. 2.0). Gene Ontology (GO) analysis was conducted using GREAT (v. 4.0.4)^70^.

### Integrative analysis of snATAC-seq and snRNA-seq

The liver snRNA data used for the integrative analysis was downloaded from GSE189600 ^17^. Cells were labeled according to the cell type information provided in the original publication. Subsequently, cells in the liver snATAC dataset were annotated by transferring cell labels from the liver snRNA dataset using tools from the Seurat (v. 5.0.2) package^71^. Utilizing the transferred labels and cell types identified through cluster-specific gene scores, correlation analyses were performed to assess the concordance between chromatin accessibility and gene expression profiles.

### Cell type-specific peaks and gene scores

Cell type-specific peaks were identified by selecting significant peaks (adjusted p-value < 0.05; log_2_ fold change > 0.58) that were not significant in more than three other cell types. Gene scores were calculated using the GeneActivity function in Signac (v1.12.9003)^68^, which quantifies the number of fragments per cell mapping to both the gene body and the promoter region (2 kb upstream).

### GWAS enrichment analysis by LDSC and SCAVENGE

The LDSC package (https://github.com/bulik/ldsc) was used to perform LDSC analysis. GWAS summary statistics were downloaded from the GWAS Catalog: Triglycerides (GCST90092955), BMI (GCST90179150), and MASLD (GCST90054782). These GWAS summary statistics were converted to a format compatible with LDSC ^72^ using the provided script (munge_sumstats.py). For each cell type-specific peak, bed files were lifted over to hg19 from hg38 to create annotation files for each cell type. Coefficient and enrichment calculations were performed by LDSC following the cell-type-specific analysis tutorial (https://github.com/bulik/ldsc/wiki/Cell-type-specific-analyses).

SCAVENGE analysis were perform as described^20^, GWAS summary statistics were downloaded from the GWAS Catalog for MASLD (GCST90091033), liver fat (GCST90016673) and cirrhosis (GCST90319878). Sentinel SNPs were identified using GCTA (v. 1.94.1)^73^ with a significance threshold of p < 10^−8^. Fine-mapping analysis was conducted using coloc (v. 5.2.3)^74^ with adj.P-value cutoff of 0.0001. The output from coloc was lifted over to hg19 from hg38 for downstream analysis. Information on counts, UMAP embeddings, and LSI embeddings was extracted from the results of the Signac analysis. Subsequently, SCAVENGE (v. 1.0.2)^20^ utilized these data to compute the trait-relevant score (TRS).

### Joint analysis of single cell motif enrichment and GWAS enrichment

Joint analysis of single-cell motif and GWAS enrichments was performed as previously described^10^, with minor modifications. Briefly, TF motif activity for each cell was quantified using chromVAR^69^, which infers TF activity by quantifying deviations in chromatin accessibility across sets of peaks sharing the same TF-binding motif. ChromVAR calculates a bias-corrected deviation score and corresponding Z-score by comparing the observed accessibility of motif-containing peaks to an expected accessibility derived from matched background peaks. Trait relevance scores (TRS) for individual cells were computed using SCAVENGE^20^. Spearman’s rank correlation coefficients were then calculated between motif activity and TRS across all cells to assess the association between transcription factor activity and GWAS trait enrichment.

### MPRA library design and construction

MASLD-associated variants were obtained from the GWAS Catalog and relevant publications (Table S1). SNPs in Linkage disequilibrium (LD) with all collected significant SNPs were identified using the LDProxy function from LDlinkR, applying thresholds of minor allele frequency (MAF) > 5% and r^2^ > 0.8 in European populations. Variants located within coding regions, as well as indels and multiallelic variants, were excluded from the analysis. For each remaining variant, a 126-base pair (bp) genomic sequence centered on the reference or alternate allele was extracted for oligonucleotide synthesis. In total, 5,369 variants were included for the MPRA library construction.

The oligonucleotide library was designed and synthetized as 170 bp oligo pool, comprising a 126 bp genomic sequence (candidate regulatory sequence, CRS) flanked by 22 bp adapter sequences at both ends (5’-GAGTACTGTATGGGCGGGTACC [126 bp oligo] GCTAGCGACAAAAGTGTCAACT-3’). The MPRA plasmid library was constructed using the pGL4.24-miniP-GFP vector, where the luciferase expression sequence in the original pGL4.24 vector was replaced with a green fluorescent protein (GFP) expression sequence. A two-step PCR was employed to incorporate a minimal TATA promoter (miniP), unique 20 bp barcodes, and the necessary vector overhang sequences into the oligonucleotide library. The oligo pool PCR products were purified and subsequently cloned into the KpnI and NcoI digested pGL4.24-miniP-GFP vector using the ClonExpress Ultra One Step Cloning Kit as described above. The ligation reactions were purified and transformed into Lucigen Endura Electrocompetent Cells (Biosearch Technologies, 60242–2) via electroporation. The resulting plasmid library was purified using the ZymoPURE II^™^ Plasmid Maxiprep Kit. Finally, CRS-barcode fragments were amplified by PCR using the primers CRS/BC_F and CRS/BC_R for subsequent sequencing to establish CRS/barcode pairings.

### Massively parallel reporter assay

The MPRA libraries were transfected into cells using Lipofectamine 3000 reagent. For HepG2 MPRA experiments, 2 × 10^7^ cells were used for one replicate. 16 h post-transfection, the medium was refreshed with complete culture medium with or without PAOA treatment and incubated for another 24 h. For LX-2 MPRA experiments, 1 × 10^7^ cells were used for one replicate. 6 h post-transfection, the medium was refreshed with complete culture medium with or without TGFβ treatment and incubated for an additional 36 h. A total of four biological replicates were performed for each experiment.

Cells were harvested and performed DNA and RNA extraction using the Quick DNA/RNA Miniprep Kit following the manufacturer’s protocol. RNA samples are rigorous DNase treated using the RNase-free Recombinant DNase I Set. For each biological replicate, 240 μg of RNA was used to synthesize the first-strand cDNA library using SuperScript II in combination with a GFP mRNA-specific primer (MPRA_BCshort_R). To amplify random barcode from plasmid DNA or cDNA, and incorporate required P5/P7 flowcell sequence and sample index, the first-round PCR was performed using the primers MPRA_BClong_P5i5_F and MPRA_BClong_P7i7_F, followed by the second-round PCR using the primers P5 and P7. Each PCR reaction utilized 48 μg of DNA or total cDNA per biological replicate as the template. All PCR reactions were carried out using the NEBNext^®^ High-Fidelity 2X PCR Master Mix. DNA and RNA barcode libraries were pooled in a 1:3 mole ratio. Library representation was assessed by next-generation sequencing on Illumina Novaseq platform.

### MPRA data analysis

MPRA plasmid library Read 1 sequences were processed by trimming the fixed sequence preceding the inserted oligo sequences using cutadapt (https://doi.org/10.14806/ej.17.1.200) with the parameters --discard-untrimmed -m 80 -g GGCCTAACTGGCCGGTACCCTGAGTACTGTATGGGCGGGTACC, retaining only the subsequent sequences corresponding to the synthesized oligos and discarding reads shorter than 80 bp. Similarly, Read 2 sequences were trimmed to remove the fixed sequence preceding the inserted barcode using cutadapt with parameters --discard-untrimmed -l 20 -g TCACCATGGTGGCTTTACCAACAG, extracting the following 20 bp as barcode sequences. The first 107 bp of each synthesized oligo were extracted to create a reference genome, and Read 1 sequences were mapped to this reference using bwa mem^75^ with parameters -L 100 -k 8 -O 5. Reads with a mapping score below 0.95 (mapping score = matched bases / total bases) were removed. Paired Read 2 sequences from the plasmid library corresponding to the retained Read 1 were used to construct oligo-barcode pairs, with duplicate pairs and barcodes mapping to multiple oligos further filtered to ensure unique pairings. For both cDNA and DNA libraries, fixed sequences were trimmed from the beginning of Read1 using cutadapt with parameters --discard-untrimmed -l 20 -g CTTGGCAATCCGGTACTTAGCGCATCTAAC. The reverse complement of the subsequent 20 bp sequences was treated as barcode sequences, and only barcodes present in the previously identified unique oligo-barcode pairs were retained for downstream analysis.

Both DNA and cDNA barcodes in each oligo-barcode pair were quantified, and barcodes with a DNA barcode count of zero were excluded to ensure data quality. To assess oligo activity, we modified the MPRAprofiler protocol as described ^76^, summarizing the total barcodes associated with each oligo for subsequent analyses. Potential enhancers were identified using DESeq2^77^ to compare cDNA and DNA barcode counts for each oligo, with oligos exhibiting an adjusted p-value < 0.05 and a log_2_ fold change > 0 (cDNA/DNA) classified as enhancers. Variants with either allele identified as active enhancers were further analyzed to determine differences in regulatory activity between the reference and alternative alleles. To detect differentially active variants, we first calculated the count ratio between the reference and alternative alleles for all variants. Subsequently, using the median ratio of four DNA replicates as a control, we normalized all RNA ratios and performed a Student’s *t*-test to calculate p-values. The Benjamini-Hochberg method was applied to adjust the p-values for multiple testing, and variants with an adjusted p-value < 0.01 were designated as significant differentially active variants.

### DAVs functional annotations

DAVs were annotated to different genomic regions using ChIPseeker^78^. Additionally, published liver chromatin interaction data, eQTLs, and ChromHMM data were downloaded to elucidate the functional significance of DAVs. Overlaps between DAVs and chromatin loops were identified using the following datasets: (1) right lobe liver intact Hi-C data from ENCODE^79^ (ID ENCFF961GKA); (2) HepG2 intact Hi-C data from ENCODE (ID ENCFF264RQT); (3) HepG2 POLR2A ChIA-PET data from ENCODE (ID ENCSR857MYZ); (4) capture-C data of promoters and H3K4me3/H3K27ac peaks in liver HepG2 cells^33^; and (5) liver promoter capture Hi-C data from GSE86189^34^, including promoter-other interactions. Each loop anchor was extended by a fixed 2.5 kb on either side. DAVs with significant variant-gene pairs were annotated using liver eQTL data from GTEx v8^80^. Chromatin states of DAVs were predicted using the core 15-state model for human liver-related tissues and cells, as determined by ChromHMM^81^ and downloaded from the ROADMAP Epigenetics Project^82^ (IDs E066, E118). Eleven of these states were considered active and used for DAV annotation: 1_TssA, 2_TssAFlank, 3_TxFlnk, 4_Tx, 5_TxWk, 6_EnhG, 7_Enh, 8_ZNF/Rpts, 10_TssBiv, 11_BivFlnk, and 12_EnhBiv. The Bedtools^83^ intersect command was utilized to calculate the overlap between the centers of DAVs and loop anchors, eQTLs, and ChromHMM-defined chromatin states.

### Enrichment of DAVs in cell type peaks

To determine whether DAVs are preferentially associated with specific cell types, we assessed the overlap between the DAVs and cell type-specific peaks derived from scATAC-seq. This analysis involved comparing DAV overlaps with cell type-specific peaks against controls generated from 1,000 iterations of randomly selected genomic regions. Fisher’s exact test was employed to calculate p-values, determining the statistical significance of the observed overlaps. Additionally, we compared the number of overlaps between DAVs and all tested variants within specific cell type peaks to evaluate the enrichment tendency of DAVs relative to non-functional variants.

We also analyzed the enrichment of all SNPs assayed by MPRA across promoter and enhancer elements in different cell types. Peaks located within promoter-proximal regions (−2 kb to +0.5 kb relative to transcription start sites) were classified as promoters, while all other peaks were designated as enhancers.

### Transcriptional factor binding analysis

Transcription factor binding site (TFBS) disruptions by DAVs were assessed using SNP2TFBS and motifBreakR^84,85^. In the SNP2TFBS analysis, statistical significance was determined by comparing the proportion of DAVs predicted to disrupt a given TF motif to the proportion of background SNPs affecting the same motif. In contrast, the motifbreakR approach assessed significance by comparing the proportion of DAVs disrupting a TF motif to the proportion of all MPRA-tested SNPs with predicted effects on that TF. TFBS with an adjusted p-value < 0.01 were considered significantly affected by DAVs. Additionally, a more permissive approach was implemented to identify potentially disrupted TFBS by individual DAVs. This involved extracting the alternative and reference sequences centered on each variant, spanning ± 20 bp (totaling 41 bp). Motifs within these variant sequences were identified using MEME’s FIMO^86^ against the HOCOMOCOv11_core_HUMAN database and HOMER TF database^87^, retaining only those motifs that encompassed the SNP location regardless of strand orientation. Subsequently, the position frequency matrix (PFM) values at the SNP position were compared between the reference and alternative alleles. Motifs with either the reference or alternative PFM value exceeding 0.3 and a ratio greater than 2 were designated as potentially interrupted motifs. For the exploration of differentially enriched TF motifs in DAVs and enhancers across various cell types and states, HOMER was utilized to identify motifs within condition-specific enhancers and DAVs, using other condition-specific enhancers or DAVs as background controls. Only motifs with a q-value < 0.1 were deemed enriched in DAVs and enhancers.

For the significantly disrupted TFs by DAVs either in HepG2 or LX-2 cells, under both control and PAOA or TGFβ treatment, we constructed protein-protein interaction network by STRING^88^ (https://cn.string-db.org/) with default parameter. Functional enrichment of these networks also displayed in WikiPathways with default parameter by STRING.

### Cell type-specific and stimuli-dependent enhancer and DAVs analysis

Enhancers were identified separately across different cell types and states using DESeq2^77^ by comparing cDNA and DNA barcode counts. Barcode counts for reference and alternative alleles were merged for each variant. Variants with an adjusted p-value < 0.05 and a log_2_ fold change > 0 were classified as significant enhancers. To identify stimuli-dependent enhancers, only enhancers detected in either control or stimulation states were considered. Firstly, we compared the cDNA/DNA ratio of control versus stimulation in enhancers, normalizing by the median control ratio for each enhancer. P-values were corrected using the Benjamini–Hochberg method, and enhancers with a corrected p-value < 0.05 were designated as stimuli-responsive enhancers. Cell type-specific enhancers were identified using the same approach. DAVs identified across different cell types or states were overlapped; DAVs detected exclusively in one cell type were considered cell type-specific DAVs, and the same criterion was applied for different stimulation states.

### CROP-seq library design and experiments

The CRISPR inhibition library (CROPi-DAVs) was designed and cloned as below. In brief, we designed sgRNAs to target 20 selected plausibly causal noncoding variants from our HepG2-MPRA screen (Supplementary Table 6). We extended 150 bp around center of each variant, then used FlashFry v1.15^89^ and CRISPick^90^ to detect potential sgRNAs. Finally, each variant was targeted by two sgRNAs from tops ranking by Hsu-Scott score. In addition, we included 6 non-targeting sgRNAs from the GeCKO v2 library as negative controls. All the sgRNAs sequences are shown in Supplemental Table S9.

To create the CROPi-DAVs plasmid library, each sgRNAs oligos were annealed, and mixed together as an oligo duplexs pool. Then the oligo pool was cloned into BsmBI-v2 (NEB, R0739L) cut lenti-CROPseq-puro-v2 backbone. The purified ligation product was transformed into Competent-Cell Stbl3 (KTSM110L). Bacterial colonies were scraped in the following day, plasmids were isolated using the Endo-Free Plasmid Midi kit (CWBIO, CW2105S). For plasmid library QC, sgRNA sequences are amplified in a single 16-cycles PCR reaction with specially designed primers (CROP-P5i5-F and CROP-P7i7-R). Library representation was determined by Next-generation sequencing (Illumina).

The CROPi-DAVs plasmid library was packaged into lentivirus as below. CROPi-DAVs plasmid library was co-transfected into HEK-293T cells with the packaging plasmid mix (psPAX2 and pMD2.G). Lentivirus was collected at 48h post-transfection, following by estimating multiplicity of infection (MOI) by counting cell survival ratio upon puromycin selection (6 μg/ml) in the HepG2 cell line. For library infection, CRISPRi_HepG2 cells seeded at 30% confluency in 10-cm dish were infected with CROPi-DAVs lentivirus library at a low MOI level (~0.1) to minimize double infection. Puromycin (6 μg/ml) screening was performed at 48h post-infection and following by maintaining for another 7 days.

After puromycin selection and expansion, cells were captured by the 10X Chromium Controller using Chromium Next GEM Single Cell 3 Reagent Kits v3.1 (10X Genomics, PN-1000268). Sample prep was performed according to protocol, holding 20~30 ng full-length cDNA per lane for sgRNA-enrichment PCR. Multiple PCR reactions were performed for sgRNA enrichment. In brief, in the first PCR reaction, full-length cDNA mix was used as template and 1st_erPCR_F and 1st_erPCR_R were used as primers. Then the first PCR product was cleaned up and amplified using the primers (2nd_erPCR_F and 2nd_erPCR_R). The final PCR was prepared in which 3rd_erPCR_F and 2nd_erPCR_R were used as primers. All PCR reactions were carried out for 10 cycles using 2x KAPA HiFi HotStart ReadyMix (KAPA Biosystems, KK2602) with annealing temperature at 62°C and 15 s extension per cycle. The whole-transcriptome scRNA-Seq libraries and the sgRNA-enrichment libraries were separately sequenced using novaXplus platform.

### CROP-seq analysis

Cell-barcode assignment in the barcode-enrichment library was performed as previously described^91^. In brief, to map sgRNAs alongside other genomic transcripts in individual cells, we constructed a custom reference genome by adding pseudo-genes representing sgRNA-containing 500 bp transcripts to the human genome assembly (Ensembl GRCh38 release). Each pseudo-gene included one sgRNA sequence, with 250 bp upstream of the sgRNA containing a U6 promoter and 230 bp downstream containing LTR sequences. Both the genome sequence file and transcription GTF file were modified synchronously to create the final reference genome for the Cell Ranger pipeline. The barcode-enrichment library was sequenced with a 150 bp paired-end (150PE) strategy, where sgRNA-containing sequences were located in Read 2 (R2) and cell barcodes along with UMIs were in Read 1 (R1). To meet the file format requirements of the 10X Genomics Cell Ranger pipeline, the first 98 bp of R2 and the first 28 bp of R1 were retained using cutadapt (https://doi.org/10.14806/ej.17.1.200). Then, sequences were mapped to the custom reference genome using cellranger (10x Genomics, v.7.1.0). Subsequently, we utilized the get_barcodes.py script^91^ to assign cell barcodes to sgRNAs. Initially, a whitelist of sgRNAs used in our library was created to comply with the requirements of get_barcodes.py. The script was then executed with the parameters --all_reads --search_seq CTTGTGGAAAGGACGAAACACCG, where the search sequence corresponds to the fixed sequence preceding the sgRNA. sgRNA targets were assigned to each cell based on dominance, requiring that a sgRNA be three times more abundant than the sum of all other sgRNAs within a cell; only unique assignments were retained. For the six lanes of single-cell transcription data from CROP-seq, each lane was individually mapped to the custom reference genome using cellranger (10x Genomics, v.7.1.0), and the resulting data were aggregated into a single dataset. The aggregated single-cell RNA-seq (scRNA-seq) data were processed using Seurat (v.4.2.0)^90^, with doublets removed by DoubletFinder (v.2.0.4)^92^. High-quality cells were retained following subsetting with criteria of nFeature_RNA > 200, nFeature_RNA < 10,000, and percent.mt < 10. The SCT normalization method was applied, and differentially expressed genes (DEGs) between sgRNA-targeting cells and other cells were identified using the FindMarkers function with the t-test method, utilizing the SCT assay and scale.data slot. Parameters were set to logfc.threshold = 0, min.cells.feature = 1, min.cells.group = 1, and min.pct = 0 to maximize the detection of potential DEGs.

### Target genes nomination analysis

To explore the numerous potential target genes of DAVs, we employed four distinct methods that consider linear distance, chromatin interactions, co-accessibility, and gene expression effects. Firstly, proximal genes were identified by selecting genes whose transcription start sites (TSS) were located within 20 kb of DAVs. Secondly, utilizing the previously described chromatin interaction data, we identified loop target genes by extending each loop anchor to 5 kb. Genes with promoters (TSS ±500 bp) located within one anchor and DAVs situated within the other anchor of the same loop were classified as loop target genes. Specifically, for liver promoter capture Hi-C and H3K4me3/H3K27ac Capture-C data, which provided gene information within one anchor, DAVs were required to reside within the other anchor to be considered loop targets. Thirdly, we leveraged the run_cicero and generate_ccans functions from the Signac package (v.1.8.0)^68^ to identify co-accessible sites from scATAC-seq data. Co-accessible sites with a score greater than 0.3 were retained, and each anchor was extended to 2 kb from the center. Genes with TSS located within one anchor and DAVs within the opposing anchor of the same co-accessible site were designated as DAVs’ co-access genes. Finally, genes associated with DAVs through significant variant-gene pairs from liver eQTL data were identified as DAV eQTL genes. These four approaches collectively considered linear proximity, chromatin interactions, co-accessibility of regulatory regions, and gene expression influences to comprehensively predict target genes regulated by DAVs.

### Inferring the gene regulatory network (GRN)

The gene regulatory network (GRN) was implemented utilizing Pando (v1.0.0)^24^. Liver snRNA-seq^17^ and snATAC-seq datasets were first integrated using Signac to enable joint modeling of gene expression and chromatin accessibility. GRNs were inferred separately for MASLD and normal hepatocytes to characterize disease-associated regulatory rewiring. The infer_grn() function in Pando was applied with default parameters, using all accessible chromatin regions and expressed genes as input features. To identify transcription factor (TF)-based regulatory modules, the find_modules() function was employed to scan all accessible regions for TF-binding motifs based on the curated motif database embedded in Pando. Peaks containing TF motifs were linked to nearby genes using the “Signac” method, and the regulatory model was fitted using the “glm” method. TF modules were subsequently constructed with default settings, and only the highest-confidence interactions for each TF were retained.

Visualization of the reconstructed GRN was performed using the NetworkGraph() function, and networks were rendered with ggraph (v2.2.2.9000). In the network visualization, node color represents the log_2_ fold change in gene expression between MASLD and normal hepatocytes, node size reflects PageRank centrality (a measure of network importance), and edge color encodes the statistical significance of each regulatory connection within its assigned module.

To delineate regulatory relationships associated with disease-associated variant (DAV) target genes, a comprehensive GRN was first reconstructed using all accessible regions and genes across cell types, followed by extraction of a DAV-focused subnetwork containing only regulatory connections linked to predefined DAV target genes.

We further profiled TF expression dynamics and motif activity within hepatocyte GRNs. TF expression levels in MASLD and normal hepatocytes were obtained using the aggregate_matrix() function in Pando. Motif activity was computed by first annotating motifs with the AddMotifs() function from Signac, followed by activity quantification using the RunChromVAR() function. Average motif activity for each TF was obtained using the wilcoxauc() function and compared between MASLD and normal states to identify differentially active TFs within the reconstructed GRN.

### Luciferase reporter assay

CRS oligos consisting of reference/alternative (ref/alt) pairs were selected based on our MPRA screen. Each 350 bp CRS sequence was synthesized as a gBlock and subsequently cloned into the pGL4.24 firefly luciferase reporter vector (Promega, E8421). Standard luciferase reporter assays were performed in HepG2, LX-2, Huh7 or HEK 293T cells. 36 h post-transfection, luciferase activity was quantified using the Dual-Luciferase^®^ Reporter Assay System according to the manufacturer’s instructions. Luminescence signals were measured in a white 384-well plate using a BioTek Synergy Neo2 plate reader. The activity of firefly luciferase was normalized to Renilla luciferase activity to assess the functional activity of the tested regulatory elements.

### CRIPSR deletion, inhibition and activation

For CRIPSR deletion sgRNA (Exci-sgRNA) plasmid construction, sgRNAs targeting the variants of interest were designed using CRISPOR. Exci-sgRNA oligos were annealed and ligated into the lentiCRISPR-v2-Puro vector or lentiCRISPR-v2-Blast vector, respectively. For CRIPSR inhibition (CRISPRi) and activation (CRISPRa) sgRNA plasmid construction, sgRNAs targeting the variants of interest were designed using CRISPick, the sgRNAs oligo inserts were annealed and cloned into the lenti-CROPseq-puro-v2 vector as described above. The lentivirus particles were produced, harvested and stored in aliquots at −80°C until use.

For CRIPSR deletion, HepG2 cells were infected with Exci-sgRNA lentivirus and selected for 7 days using puromycin (6 μg/ml) and blasticidin (30 μg/ml). The deletion effect of each targeted variant was estimated by PCR amplification using primers targeting outside of the deletion sequence (Supplemental Table S9). The deleted small genomic regions were detected using agarose gel electrophoresis. For CRISPR inhibition, the CRISPRi_HepG2 cells were infected with CRISPRi-sgRNA lentivirus followed by puromycin selection (6 μg/ml) for at least 7 days. For CRISPR activation, the CRISPRa_HepG2 cells were infected with CRISPRa-sgRNA lentivirus followed by puromycin selection (6 μg/ml) and doxycycline treatment (5 μg/mL) for at least 7 days. All the sgRNAs sequences and PCR amplification primers are shown in Supplemental Table S9.

### Gene editing in HepG2

PE5b system was used for SNP editing in HepG2 following the protocol as previously described^93^. The epegRNA and nickRNA was designed to recognize the surrounding region of the SNP using PRIDICT2.0 (https://www.pridict.it/), then the epegRNA dsODN for rs474513(G>A) and nickRNA dsODN for rs474513 were synthetized and cloned into tevopreq1 epegRNA vector and nick-sgRNA vector respectively. The rs474513(G>A) epegRNA plasmid, rs474513 nick-sgRNA plasmid, pCMV-PEmax-P2A-GFP and pEF1a-MLH1dn were co-transfected into HepG2 using Lipofectamine 3000 reagent. 48 h post-transfection, the GFP-positive cells were sorted by FACS and the subclones were identified by Sanger sequencing.

### RT-qPCR

Cells were collected and RNA was isolated with RNeasy Mini Kit according to the manufacturer’s instructions. For mRNA detection, RNA samples were reverse transcribed using HiScript^®^ III RT SuperMix for qPCR (+gDNA wiper), and qRT-PCR was performed with ChamQ Universal SYBR qPCR Master Mix using Applied Biosystems QuantStudio 5 (Thermo Fisher Scientific, USA). Sequences of the qPCR primers are shown in Supplemental Table S9. Relative quantification analysis was performed using the ΔΔCt method, with *HPRT1* as an endogenous reference. Relative gene expression is presented as the ratio of the target gene to reference control.

### Hepatic-like cells differentiation

The monoclonal human iPSC lines were differentiated to hepatic-like cells as previously described^94^. In brief, hiPSCs were differentiated into Definitive Endoderm (DE) using STEMdiff Definitive Endoderm Kit for 4 days following the manufacturer’s instructions. For 4–9 days, cells were cultured in Advanced DMEM/F12 with 1% B27 (Cat: #A1895601), 1% KSR, 1% Glutamax, 1% MEM-NEAA, 0.5 μM A83–01, 0.25 μM Na-butyrate and 0.5% DMSO. Cells were maintained at 37°C in 5% CO_2_ with 95% air and the medium was replaced daily. For 9–16 days, cells were cultured in Advanced DMEM/F12 with 1% B27 (Cat: #A1895601), 1% KSR, 1% Glutamax, 1% MEM-NEAA, 0.5 μM A83–01, 15 μM FH1, 15 μM FPH1, 0.1 μM Dexamethasone and 10 μM Hydrocortisone. Cells were maintained at 37°C in 5% CO_2_ with 95% air and the medium was replaced daily. Cells were collected at Day0, Day4, Day9, Day14 and Day16, and lysed for RNA extraction and RT-qPCR detection.

### Hepatic organoid (HLO) differentiation

The hepatic organoid (HLO) differentiation was performed as previously described^95^. In brief, hiPSCs were differentiated into Definitive Endoderm (DE) using STEMdiff Definitive Endoderm Kit for 4 days following the manufacturer’s instructions. For 4–6 days, cells were cultured in Advanced DMEM/F12 with 2% B27 (Cat#17504044) and 1% N2 containing 500 ng/mL fibroblast growth factor 4 (FGF4) and 3 μM CHIR99021. Cells were maintained at 37°C in 5% CO_2_ with 95% air and the medium was replaced daily. The FG cells were detached by Accutase and suspended with Matrigel matrix on ice. The mixture of cells and Matrigel was embedded in 30 μL drops on dishes in Advanced DMEM/F12 with 2% B27, 1% N2, 10 mM HEPES, 1% Glutamax, 1% Pen/Strep, 5 ng/mL fibroblast growth factor 2 (FGF2), 10 ng/mL vascular endothelial growth factor (VEGF), 20 ng/mL epidermal growth factor (EGF), 3 μM CHIR99021, 0.5 μMA83–01, and 50 mg/mL ascorbic acid, and incubated in the CO_2_ incubator for 4 days with medium changed every 2 days. The medium was then switched to Advanced DMEM/F12 with 2% B27 (Cat#17504044), 1% N2, 10 mM HEPES, 1% Pen/Strep, and 2 μM retinoic acid (RA), and incubated for further 4 days with medium changed every 2 days. The final media switch was to the hepatocyte culture medium (HCM) and the cells were incubated for another 6 days, changing the medium every 2 days.

### Fatty liver organoid HLOs model

Modeling of steatohepatitis in HLOs was performed as previously reported^95^. HLOs was isolated from Matrigel and washed with cold PBS in 4°C for three times, then cultured with HCM media containing 5 μg/mL insulin and 300 μM oleic acid and 150 μM palmitic acid on ultra-low attachment 6 multi-well plates (Corning) to induce steatohepatitis. HLOs were collected at day 4 for immunostaining and RNA isolation. Accumulation of lipid in HLOs was measured using BODIPY 493/503.

### 3D Liver Spheroids

The HepG2/LX-2 spheroids were generated following the protocol described previously^49^. HepG2-Exci-sgRNA stable cell lines and LX-2 cell lines were mixed in a 24:1 ratio into 96-well round-bottomed ultra-low attachment plates (Corning) at 2000 cells/well in 100 μL minimum essential medium supplemented with 10% fetal bovine serum (FBS). After 72 h, they were treated with 300 μM oleic acid and 150 μM palmitic acid for another 48h.

### Immunostaining

For HepG2 cell lines and HepG2/LX-2 3D spheroids immunostaining, cells were washed twice with PBS and fixed with 4% Paraformaldehyde (PFA) for 15min at room temperature. After this, samples were stained with 5μM BODIPY (493/503 nm) and 1 μg/ml DAPI for 15 min at 37°C.

For LX-2 cell lines immunostaining, cells were washed twice with PBS and fixed with 4% Paraformaldehyde (PFA) for 15 min at room temperature. After this, samples were then permeated with 0.1% Triton X-100/PBS for 5 min and blocked with 10% BSA/PBS to avoid nonspecific staining for 1 h. Samples were reacted with primary antibodies diluted in 5% BSA at 4°C overnight, following by incubation with fluorophore-labeled secondary antibodies (4 μg/mL) diluted in 5% BSA/PBS at 37°C for 30 min, followed by staining DAPI (1 μg/mL) diluted in PBS at room temperature for 15 min.

For HLOs immunostaining, HLOs were collected and washed twice with PBS and fixed with 4% PFA for 2 h at room temperature. Then, HLOs were permeabilized with 0.5% Triton X-100/PBS for 20 min at room temperature and blocked with PBS containing 0.5% Triton X-100 and 1% bovine serum albumin (BSA) for another 2 h at room temperature. HLOs were then stained with specific antibodies or 5 μM BODIPY (493/503 nm) and 1 μg/ml DAPI. After with washing with three times of DPBS containing 0.5% Triton X-100 and 0.2% BSA, HLOs were ready for imaging. All samples were imaged using confocal microscope (Nikon A1).

### FACS

HepG2 cell lines were detached by trypsin and wash with PBS for 3 times, following by 4% PFA fixation for 15 min at room temperature. After this, samples were stained with 5 μM BODIPY (493/503 nm) and 1μg/ml DAPI for 1.5 h at 37°C. Cells were washed in PBS and resuspended in PBS and transferred into Test Tube with Cell Strainer Snap Cap. Analysis of cells was performed using Agilent Novocyte Advanteon.

### Polygenic risk score analysis

The PRS analysis was conducted using available data from the VA’s Million Veteran Program (MVP)^60^ and initial MASLD-related analyses within VA Merit-funded MVP Cardiometabolic Project (MVP003/028)^4^. MVP is a large-scale biobank, supported by the Veterans Health Administration Office of Research and Development in the United States. The study protocols of MVP biobank and MVP Cardiometabolic Project were approved by Veterans Affairs (VA) Central Institutional Review Board (CIRB). The design, baseline demographics, and quality control procedures for MVP biobank have been previously described^60^. The MASLD-related phenotype and genetic analyses in MVP Cardiometabolic Projects formed the basis for the current PRS analyses^4^. PRS was constructed to test the MASLD predictive power of SNPs prioritized by MPRA. After removing the SNPs in linkage disequilibrium (r2 > 0.05), the 114 independent SNPs out of the 365 SNPs prioritized by MPRA assays were used to build PRS. A matched set of 114 SNPs, selected from meta-analyzed results of 10 different randomized draws of MPRA total SNPs, excluding 365 DAVs, served as the negative control. A set of 9 missense SNPs (PRS9; Table S8), which are the well-established MASLD causal SNPs^4^, served as the positive control.

Four different MASLD phenotype proxies were used to identify MASLD: MASLD-ALT, MASLD-ICD, ALT and FIB4 max progression. (1) For MASLD-ALT, MASLD cases were defined as participants with elevated ALT (> 40 U/L for men or >30 U/L for women during at least two time points at least 6 months apart within a 2-year window at any point prior to enrollment and exclusion of other causes of liver disease, chronic liver diseases or systemic conditions and/or alcohol use disorders. (2) For MASLD-ICD, MASLD cases were identified by ICD9 codes (456.2, 456.21, 571.5, 572.2 and 572.3) and ICD-10 codes (K72.9, K72.91, K74.0, K74.02, K74.1, K74.2, K74.6 and K74.69). (3) The MASLD phenotype proxy, ALT, was a continuous variable representing ALT levels. (4) Fibrosis-4 (FIB4) max progression was a surrogate marker of liver fibrosis progression by estimating FIB4 trajectory over time ^96^. FIB4 was calculated using the formula ([age × AST]/[platelet × sqrt (ALT)]) as previously described. AST, ALT, and platelet count (in K/mm3) of all outpatient were collected from VA laboratories from January 2002 to November 2021.

To construct the PRS, logistic regression was performed between SNPs and MASLD proxies MASLD-ALT, MASLD-ICD, or FIB-4 max progression, while linear regression was used between SNPs and ALT levels. Variant weights were derived from effect sizes reported in the GWAS by Miao et al^7^. The regression analysis of PRS with MASLD phenotypes was performed using a linear regression model adjusted for age, sex, and principal components of genetic ancestry. The regression coefficients from these analyses represented log-odds change in the outcome for each unit of increase in the standardized PRS. To compare the predictive power in deciles, the individuals were divided into 10 deciles based on their PRS scores and assessed the odds ratio (OR) of having MASLD (MASLD-ALT) in each of the 10 deciles when compared to the lowest decile.

### Comparison of DAV-derived PRS with published PRS models

The polygenic risk score (PRS) constructed from disease-associated variants (DAVs) was benchmarked against the best-performing published MASLD PRS (PGS002732) derived from the MVP cohort, as well as PRS9 and PGS000655^4,27^. To minimize potential overfitting, all PRS models were evaluated in the *All of Us Research Program* (AoU)as an independent validation cohort. AoU is a large, US-based biobank developed to improve and enable the inclusion of diverse, underrepresented populations in large-scale genetic and epidemiological research studies^97^. We accessed short read whole genome sequencing (srWGS) data, survey data, and electronic health record data from participants included in the v8 release of AoU. Age within AoU was calculated as participants’ age as of their consent date to AoU. Sex was determined using self-reported sex-at-birth data provided by AoU.

We used MASLD-ALT as a proxy for MASLD, as defined above. ALT labs in units per liter were extracted using LOINC code 1742–6. Measurements less than 0 were removed, then outlier measurements were removed if they were 3 standard deviations away from the population average in log space.

DAV PRS for MASLD were calculated using the 114 LD-independent DAVs and marginal effect sizes from the Vujkovic et al. GWAS^4^. Published PRS were downloaded from the PGSCatalog^98^. All PRS were calculated using PLINK v1 with the --score sum argument. We residualized global ancestry from the PRS as previously described^99^, then standardized the scores. Association analyses were adjusted for age, sex, the first 10 PCs, and sequencing site. Statistical differences in effect size were estimated using a two-sided Z-test. P-values were adjusted for multiple testing per analysis using the Benjamini-Hochberg procedure.

### Quantification and statistical analysis

Data are presented as mean ± SEM. Statistical significance was determined by unpaired two-tailed student t-test, Fisher’s exact test or two-way anova analysis using GraphPad Prism software or R software; a p-value of < 0.05 or FDR<0.01 was considered significant. Specific statistical tests performed are listed in the respective figure legends or sections of the Methods.

## Supplementary Material

Supplementary Files

This is a list of supplementary files associated with this preprint. Click to download.

• TableS1.xlsx

• TableS2.xlsx

• TableS3.xlsx

• TableS4.xlsx

• TableS5.xlsx

• TableS6.xlsx

• TableS7.xlsx

• TableS8.xlsx

• TableS9.xlsx

• Supplementaryinformation.docx

• ExtendFigures.pdf

## Data Availability

The accession number for the sequencing data reported in this paper are GSE281367, GSE281364, and GSE281160.
